# Supplementary material for: A co-assembly platform engaging macrophage scavenger receptor A for lysosome-targeting protein degradation
Source: Nat Commun. 2024 Feb 23;15:1663. doi: 10.1038/s41467-024-46130-0 (PMC10891067; doi:10.1038/s41467-024-46130-0)
Supplement: Supplementary file 1 — Supplementary Information [file 41467_2024_46130_MOESM1_ESM.pdf]

Supplementary Information

A Co-Assembly Platform Engaging Macrophage Scavenger Receptor A for Lysosome-Targeting Protein Degradation

Qian Wang[1,2,3,4], Xingyue Yang[1,2,3,4], Ruixin Yuan[1,2,3], Ao Shen[1,2,3], Pushu Wang[1,2,3], Haoting Li[1,2,3], Jun Zhang[1,2,3], Chao Tian[3], Zhujun Jiang[1], Wenzhe Li[1], and Suwei Dong[1,2,3]*

[1]State Key Laboratory of Natural and Biomimetic Drugs, Peking University, Beijing, China.

[2]Chemical Biology Center, Peking University, Beijing, China.

[3]Department of Chemical Biology, School of Pharmaceutical Sciences, Peking University, Beijing, China.

[4]These authors contributed equally: Qian Wang, Xingyue Yang.

*e-mail: dongsw@pku.edu.cn

Table of Contents

Supplementary Figures S2

Supplementary Tables..S39

Supplementary Methods..S40

Supplementary References..S44

Supplementary Figures

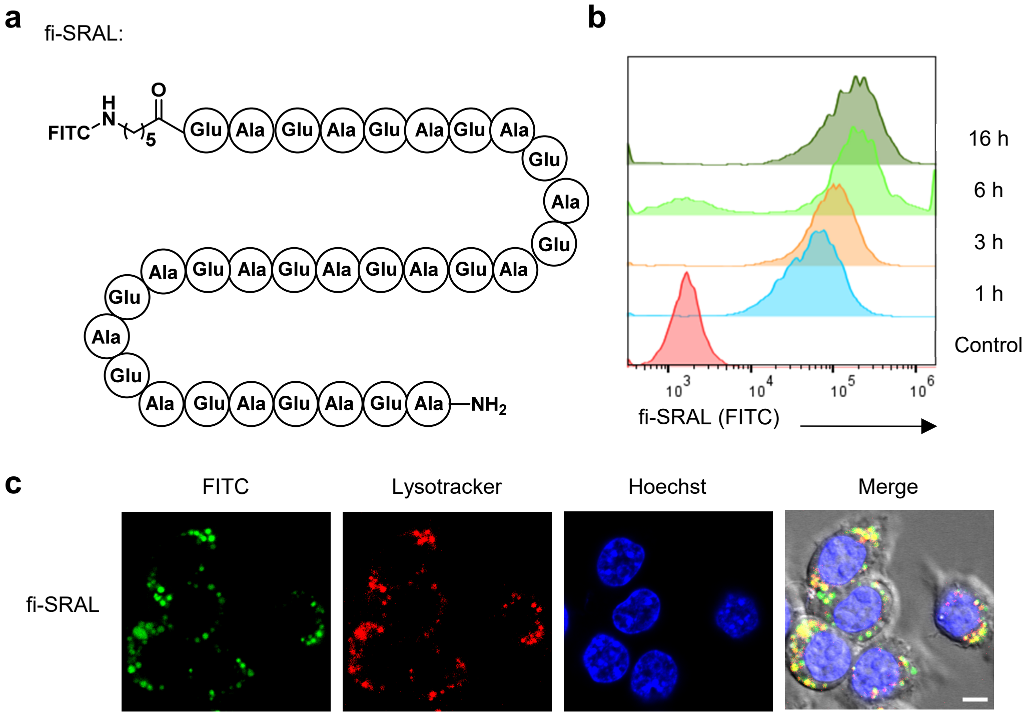

Supplementary Fig. 1 fi-SRAL can be taken up and transported to lysosome. a) Sequence of FITC-modified SR-A ligand peptide SRAL (fi-SRAL). **b)** Representative flow cytometry results (n = 3) showing that RAW264.7 cells take up the fi-SRAL in a time-dependent way and the intracellular fluorescence approach saturation at 6 h. **c)** Visualization of fi-SRAL (green) and lysosome (red) co-localization in RAW 264.7 cells by confocal microscopy. Scale bar: 5 μm.

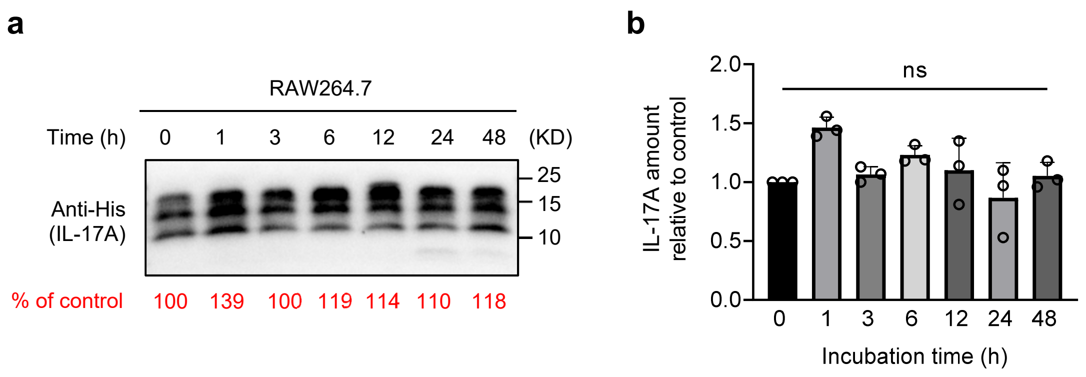

Supplementary Fig. 2 The medium IL-17A exhibited no discernible alterations, suggesting an overdose of the provided protein. a) Medium IL-17A measurement by western blot in the time-course experiment for IL-17A uptake. RAW264.7 cells incubated with 0.5 µg/ml IL-17A and 50 µM mixed fi-SRAL and fi-17Abp for 1, 3, 6, 12, 24 and 48 h. Medium IL-17A was taken at the indicated time and analyzed by western blot. **b)** The corresponding quantified result of **a)**. Densitometry was used to calculate protein levels, and data (n = 3 biologically independent experiments) were normalized to the GAPDH. P values were determined by one-way ANOVA with Tukey's multiple comparisons test. ns, no significance.

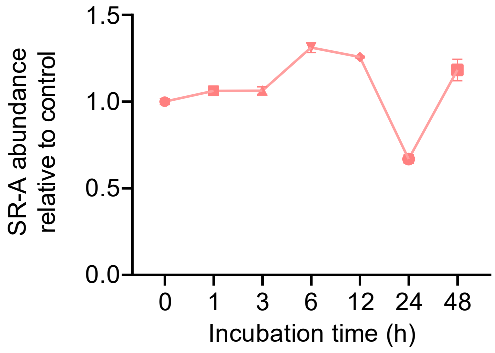

Supplementary Fig. 3 Surface SR-A undergoes recycling throughout the time-dependent degradation process. RAW264.7 cells incubated with 0.5 µg/ml IL-17A and 50 µM mixed fi-SRAL and fi-17Abp for 1, 3, 6, 12, 24 and 48 h. Cell surface SR-A was measured at the indicated time. Mean fluorescence intensity (MFI) was determined by flow cytometry and the relative MFI was normalized to the control group. Data are presented as the mean ± SD, and error bars represent the standard deviation of biological replicates (n = 3).

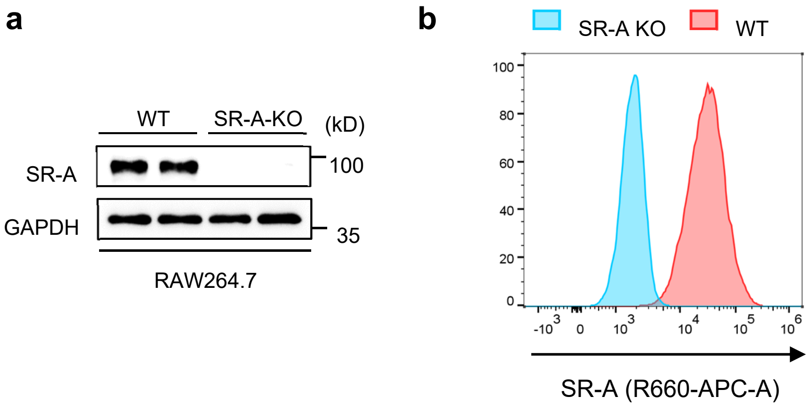

Supplementary Fig. 4 RAW264.7 cell line with stable knockout of SR-A using CRISPR/Cas9 system. a) Western blot of SR-A in wildtype (WT) and SR-A knockout (SR-A-KO) RAW264.7 cells (n = 3). **b)** Representative flow cytometry results (n = 3) of SR-A level in WT and SR-A-KO RAW264.7 cells.

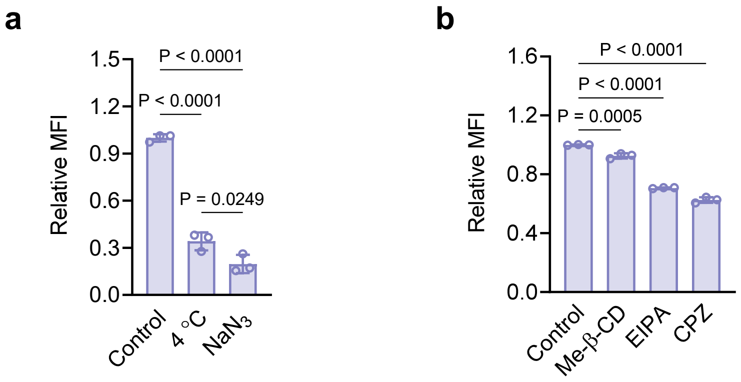

Supplementary Fig. 5 Uptake of IL-17A by macrophages is an energy-dependent process involving several endocytosis pathways. a) Uptake of IL-17A in RAW264.7 cells incubated at 4 °C or pretreated with 100 mM NaN_3 for 0.5 h followed by the addition of 0.5 μg/ml IL-17A and 50 μM mixed fi-SRAL and fi-17Abp peptides for 6 h. **b)** Uptake of IL-17A in RAW264.7 cells incubated with 0.5 mM Me-β-CD (lipid rafting pathway inhibitor), 20 μM EIPA (pinocytosis inhibitor) or 3 μg/ml CPZ (clathrin-endocytosis inhibitor) for 0.5 h followed by the addition of 0.5 μg/ml IL-17A and 50 μM mixed fi-SRAL and fi-17Abp peptides for 6 h. MFI was determined by flow cytometry and the relative MFI was normalized to the control group. Data are presented as the mean ± SD, and error bars represent the standard deviation of biological replicates ($n = 3$). P values were determined by one-way ANOVA with Tukey's multiple comparisons test.

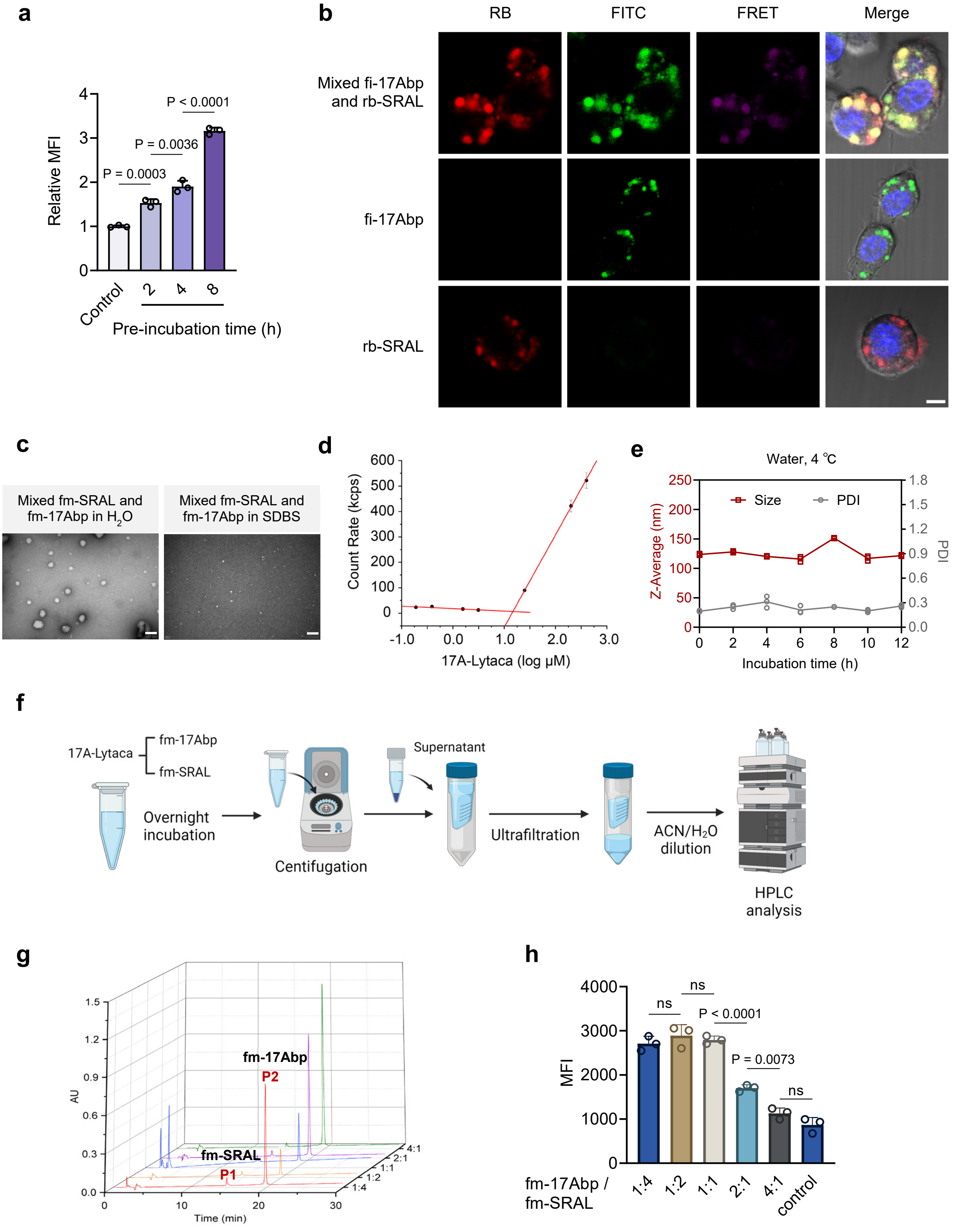

a
b
RB
FITC
FRET
Merge
Mixed fi-17Abp and rb-SRAL
fi-17Abp
rb-SRAL
Relative MFI
P < 0.0001
P = 0.0036
P = 0.0003
Control
2
4
8
Pre-incubation time (h)
c
Mixed fm-SRAL and fm-17Abp in H_2O
Mixed fm-SRAL and fm-17Abp in SDBS
d
Count Rate (kcps)
17A-Lytaca (log μM)
e
Water, 4 ℃
Size
PDI
Z-Average (nm)
Incubation time (h)
f
17A-Lytaca
fm-17Abp
fm-SRAL
Overnight incubation
Centifugation
Supernatant
Ultrafiltration
ACN/H_2O dilution
HPLC analysis
g
fm-17Abp
P2
fm-SRAL
P1
AU
Time (min)
4:1
2:1
1:1
1:2
1:4
h
MFI
ns
ns
P < 0.0001
P = 0.0073
ns
fm-17Abp / fm-SRAL
1:4
1:2
1:1
2:1
4:1
control

Supplementary Fig. 6 The molecular mechanism of mixed 17Abp and SRAL for facilitating the IL-17A endocytosis. a) Uptake of IL-17A in RAW264.7 cells after 6 h of treatment with 0.5 μg/ml IL-17A and 50 μM fi-SRAL and fi-17Abp with different pre-incubation time (2 h, 4 h, 8 h). **b)** Visualization of FRET phenomenon of peptide fi-17Abp and rb-SRAL in RAW264.7 cells by confocal microscopy. Scale bar: 5 μm. **c)** Representative TEM images (n = 3) for mixed fm-SRAL and fm-17Abp in H_2O or sodium dodecylbenzene sulfonate (SDBS, 800 μM) buffer at a concentration of 400 μM. Scale bar, 100 nm. **d)** The critical aggregation concentration (CAC) of the co-assembled complex comprising fm-SRAL and fm-17Abp (molar ratio 1:1). Peptides were incubated at 4 °C for 12 h and then detected by dynamic light scattering (DLS) for CAC calculation (n = 3). **e)** Time-dependent changes in the hydrodynamic size (red) and polydisperse index (PDI, grey) of the co-assembled supramolecular complex formed by fm-17Abp and fm-SRAL at 4 °C (n = 3). **f)** Schematic illustration of the work flow about stoichiometry determination. ACN, acetonitrile; HPLC, high-performance liquid chromatography. Created with BioRender.com. **g)** HPLC analysis of the co-assembled complex with molar ratios of fm-17Abp to fm-SRAL at 1:4 (160 μM: 640 μM), 1:2 (267 μM: 533 μM), 1:1 (400 μM: 400 μM), 2:1 (533 μM: 267 μM), and 4:1 (640 μM: 160 μM). **h)** Ratio effect assay for IL-17A uptake in RAW264.7 cells incubated with 0.5 μg/ml IL-17A and 17A-Lytaca with molar ratios of fm-17Abp to fm-SRAL at 1:4 (160 μM: 640 μM), 1:2 (267 μM: 533 μM), 1:1 (400 μM: 400 μM), 2:1 (533 μM: 267 μM), and 4:1 (640 μM: 160 μM). For **a** and **h**, MFI was determined by flow cytometry and the relative MFI was normalized to the control group. Data are presented as the mean ± SD, and error bars represent the standard deviation of biological replicates (n = 3). P values were determined by one-way ANOVA with Tukey's multiple comparisons test.

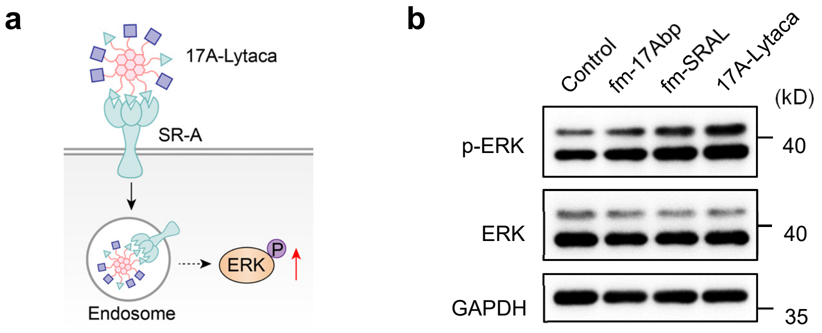

Supplementary Fig. 7 17A-Lytaca particles trigger SR-A-mediated activation of signaling responses. a) Schematic illustration of the cellular activation of ERK signaling pathway by SR-A-mediated uptake of 17A-Lytaca. **b)** Western blot of ERK and p-ERK in RAW264.7 cells treated with 50 μM 17A-Lytaca, fm-17Abp or fm-SRAL alone for 6 h (n = 3). This finding indicated cellular activation induced by 17A-Lytaca, aligning with previous studies[1, 2] where the ERK signaling pathway was implicated in SR-A-mediated uptake. ERK, extracellular regulated protein kinase; p-ERK, phospho-ERK.

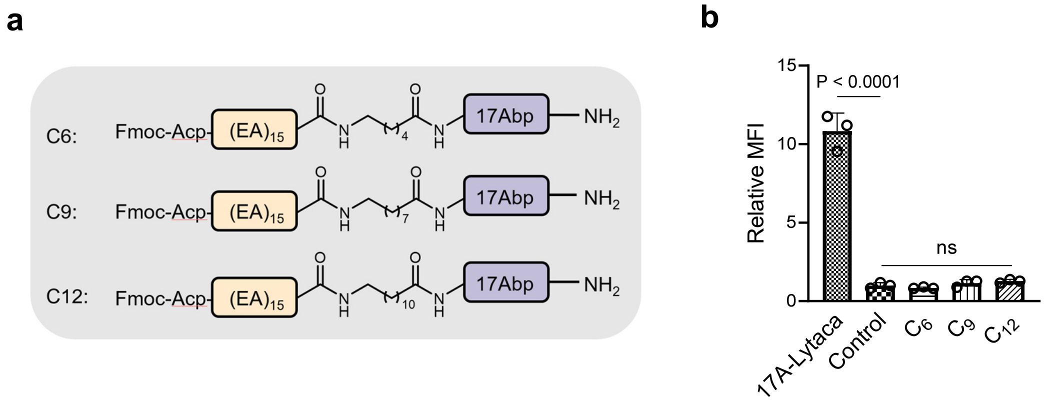

Supplementary Fig. 8 Fmoc-modified covalent peptide chimeras with different linker length cannot promote the uptake of IL-17A. a) Structures of designed covalent chimeras with various conjugate linkers. **b)** Experiment for linker length screening. RAW264.7 cells were exposed to 0.5 µg/ml IL-17A and 50 µM each of peptides C6, C9, or C12. Intracellular IL-17A signal was evaluated using flow cytometry. The relative MFI was normalized to the control group. Data are presented as the mean ± SD, and error bars represent the standard deviation of biological replicates (n = 3). P values were determined by one-way ANOVA with Tukey's multiple comparisons test.

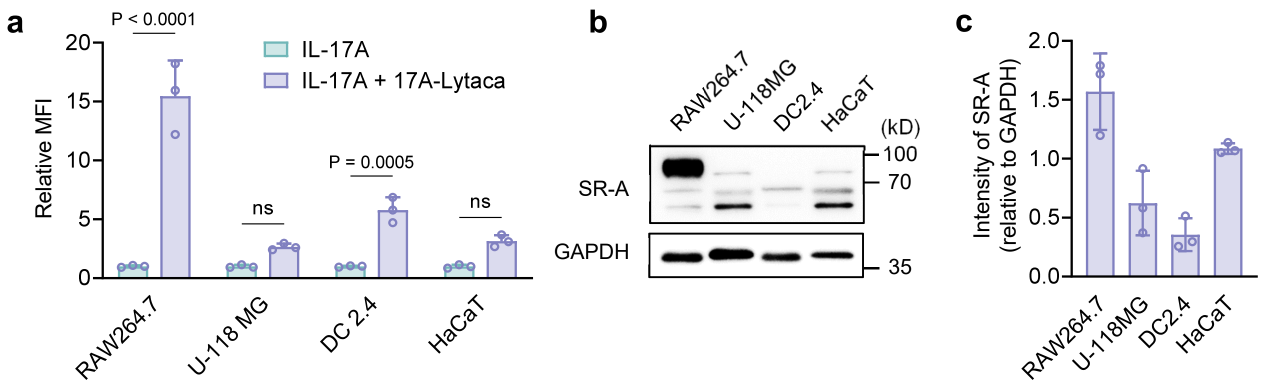

Supplementary Fig. 9 The IL-17A endocytosis in RAW264.7 cell line is higher than other cell lines. a) IL-17A levels in RAW264.7, U-118 MG, DC 2.4 and HaCaT cells after a 6 hour-treatment with 0.5 µg/ml IL-17A and 50 µM 17A-Lytaca. MFI was determined by flow cytometry and the relative MFI was normalized to the control group. Data are presented as the mean ± SD, and error bars represent the standard deviation of biological replicates (n = 3). P values were determined by two-way ANOVA with Sidak's multiple comparisons test, ns, no significance. **b)** Western blot analysis of SR-A in RAW264.7, U-118 MG, DC 2.4 and HaCaT cells and **c)** the corresponding quantified results. Densitometry was used to calculate protein levels, and data (n = 3 biologically independent experiments) were normalized to the GAPDH.

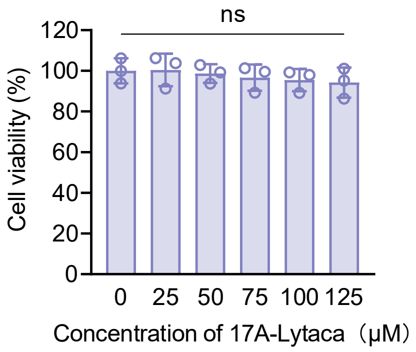

Supplementary Fig. 10 17A-Lytaca treatment shows no obvious toxicity to cells. Cell viability was measured in RAW264.7 cells treated with 0, 25, 50, 75, 100 or 125 µM 17A-Lytaca. Data are presented as the mean ± SD, and error bars represent the standard deviation of biological replicates (n = 3). P values were determined by one-way ANOVA with Tukey's multiple comparisons test. ns, no significance.

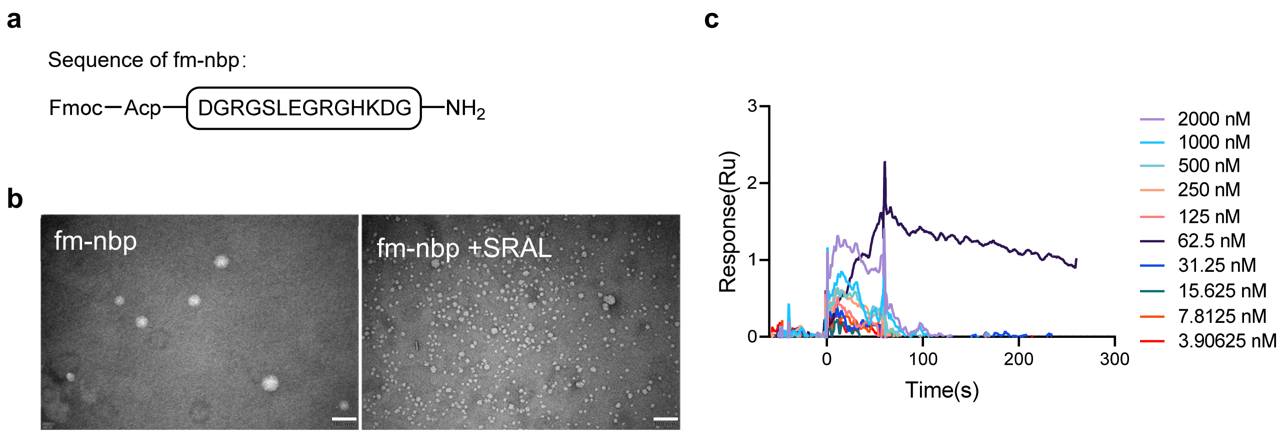

Supplementary Fig. 11 Design of control-Lytaca with fm-nbp showing no binding to IL-17A. a) Sequence of the fm-nbp. **b)** Representative TEM images for fm-nbp and control-Lytaca particles (mixture of fm-nbp and fm-SRAL) at a concentration of 400 μM. Scale bar, 100 nm. **c)** SPR curve of fm-nbp at the indicated concentration binding to mouse IL-17A immobilized on the CM5 chip surface. fm-nbp, Fmoc-modified IL-17A no binding peptide.

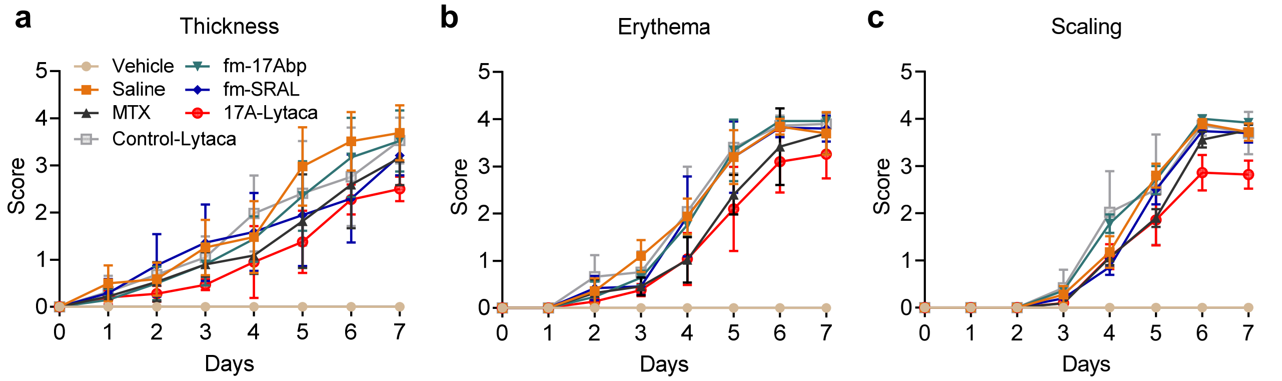

Supplementary Fig. 12 Psoriasis area and severity index (PASI) of mice. a) Epidermal thickness. **b)** Erythema. **c)** Scaling. Data are presented as the mean ± SD, and error bars represent the standard deviation of biological replicates (n = 5).

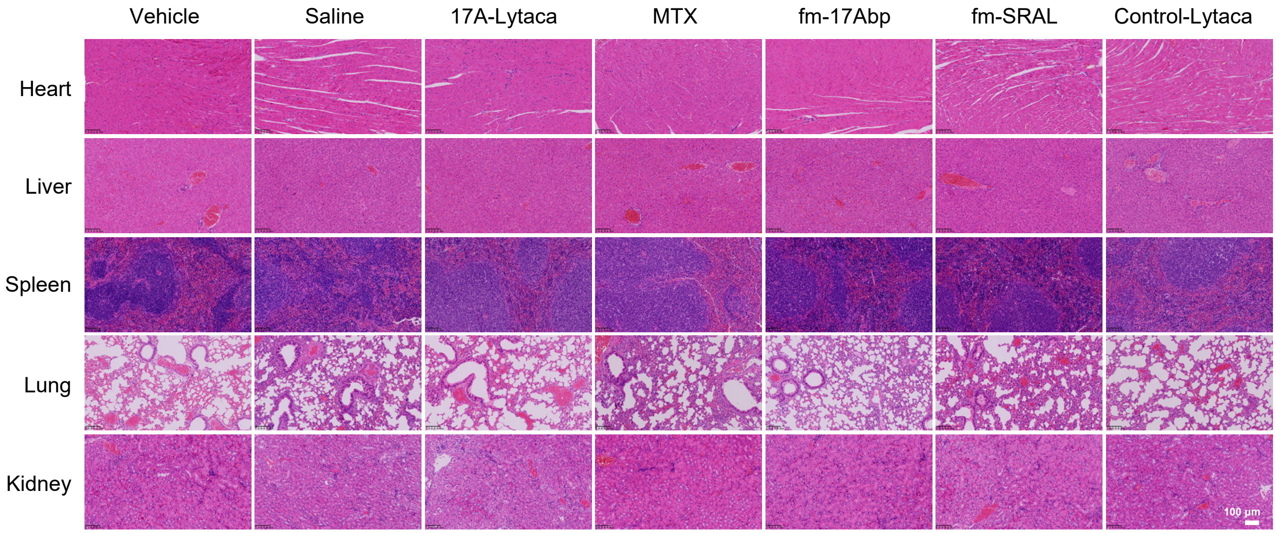

Supplementary Fig. 13 17A-Lytaca treatment have no obvious toxicity to the organs of mice. H&E staining of the heart, liver, spleen, lung, and kidney tissues in the BALB/c mice treated with saline, 17A-Lytaca, MTX, fm-17Abp, fm-SRAL or Control-Lytaca. Scale bar, 100 μm.

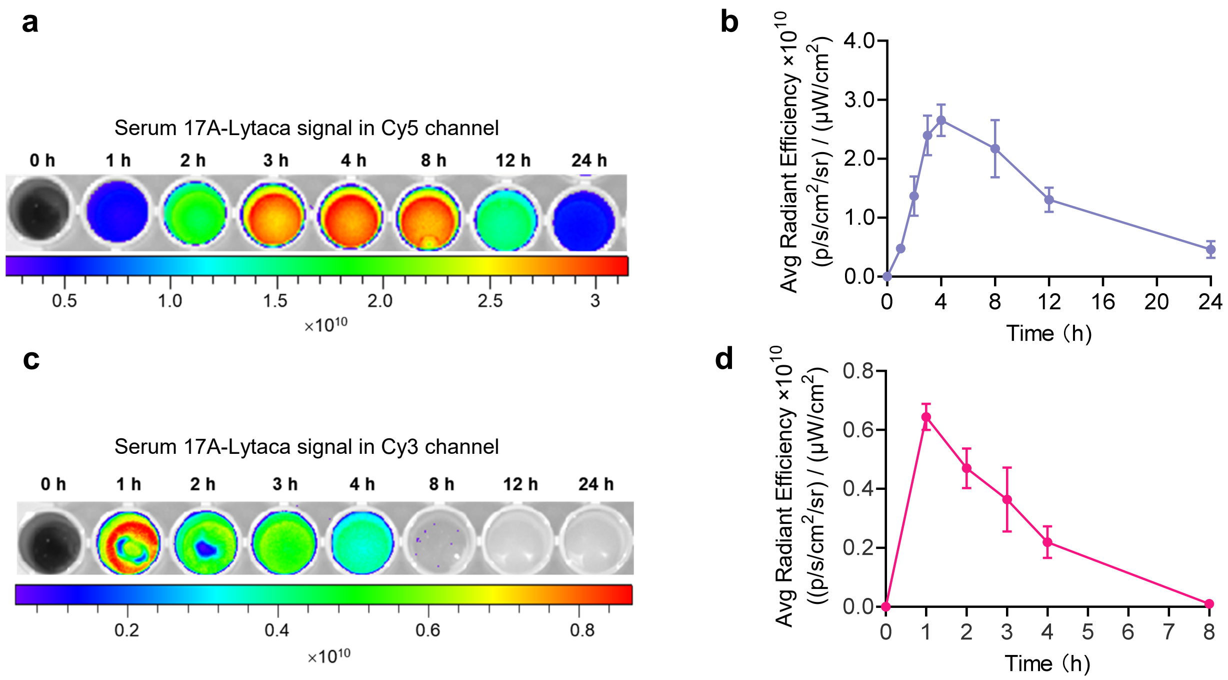

Supplementary Fig. 14 Serum clearance kinetics assay. The experiment was conducted in healthy mice with Cy-labeled 17A-Lytaca, comprising Cy5-labeled fm-17Abp (15 mg/kg) and Cy3-labeled fm-SRAL (22 mg/kg). **a)** Time-dependent fluorescence images of serum captured in Cy5 channel. **b)** Quantitative Cy5 fluorescence intensity of 17A-Lytaca. **c)** Time-dependent fluorescence images of serum captured in Cy3 channel. **d)** Quantitative Cy3 fluorescence intensity of 17A-Lytaca. Data are presented as the mean ± SD, and error bars represent the standard deviation of biological replicates (n = 3).

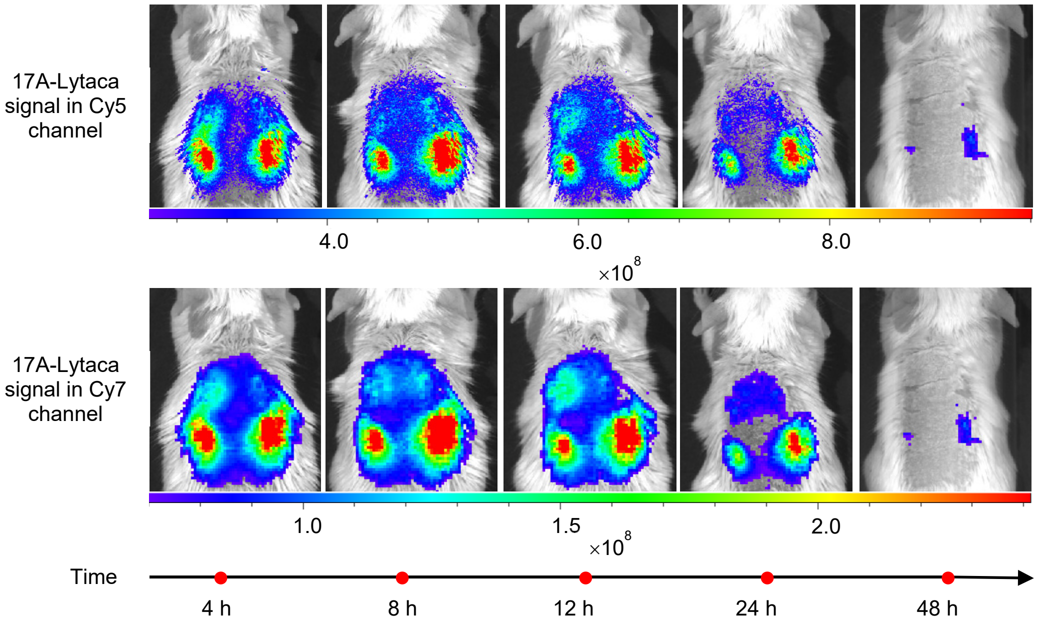

Supplementary Fig. 15 17A-Lytaca diffused in the skin lesion and exhibited prolonged *in vivo* retention in psoriasis mice. Time-dependent *in vivo* fluorescence images of psoriasis mice after intraperitoneal treatment with Cy-labeled 17A-Lytaca, comprising Cy5 labeled fm-17Abp (5 mg/kg) and Cy7 labeled fm-SRAL (7.3 mg/kg).

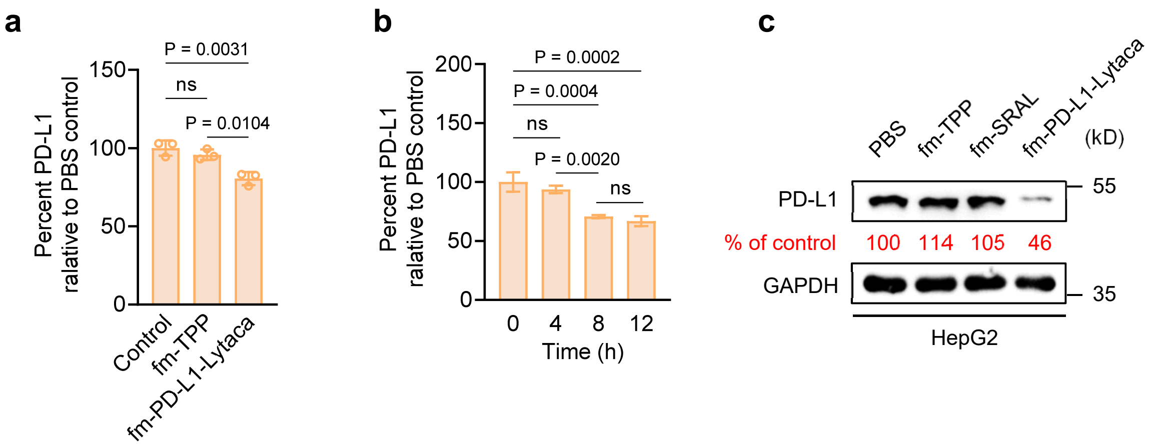

Supplementary Fig. 16 fm-PD-L1-Lytaca enable degradation of the membrane protein PD-L1. **a)** MFI relative to the control (PBS) for RAW264.7 cells incubated for 8 h with 50 μM TPP or PD-L1-Lytaca. **b)** Time course of degradation of cell surface PD-L1 in RAW264.7 cells incubated with 50 μM PD-L1-Lytaca for 0, 4, 8 and 12 h. **c)** Western blot of PD-L1 in HepG2 cells treated with 50 μM fm-TPP, fm-SRAL or fm-PD-L1-Lytaca for 12 h. Densitometry was used to calculate protein levels, and data were normalized to the GAPDH. For **a** and **b**, MFI was determined by flow cytometry and the relative MFI was normalized to the control group. Data are presented as the mean ± SD, and error bars represent the standard deviation of biological replicates (n = 3). P values were determined by one-way ANOVA with Tukey's multiple comparisons test, ns, no significance.

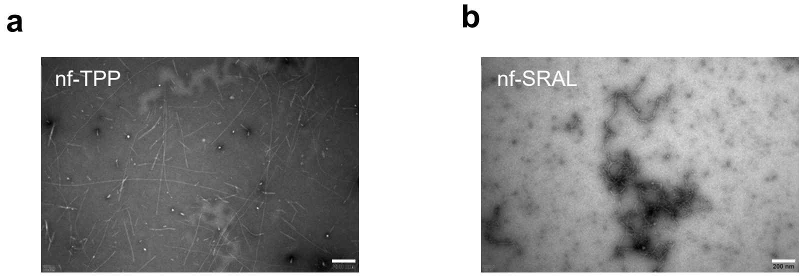

Supplementary Fig. 17 nf-TPP or nf-SRAL alone showed structure of fiber. Representative TEM images (n = 3) for **a)** nf-TPP and **b)** nf-SRAL in H_2O at a concentration of 400 μM. Scale bar, 200 nm.

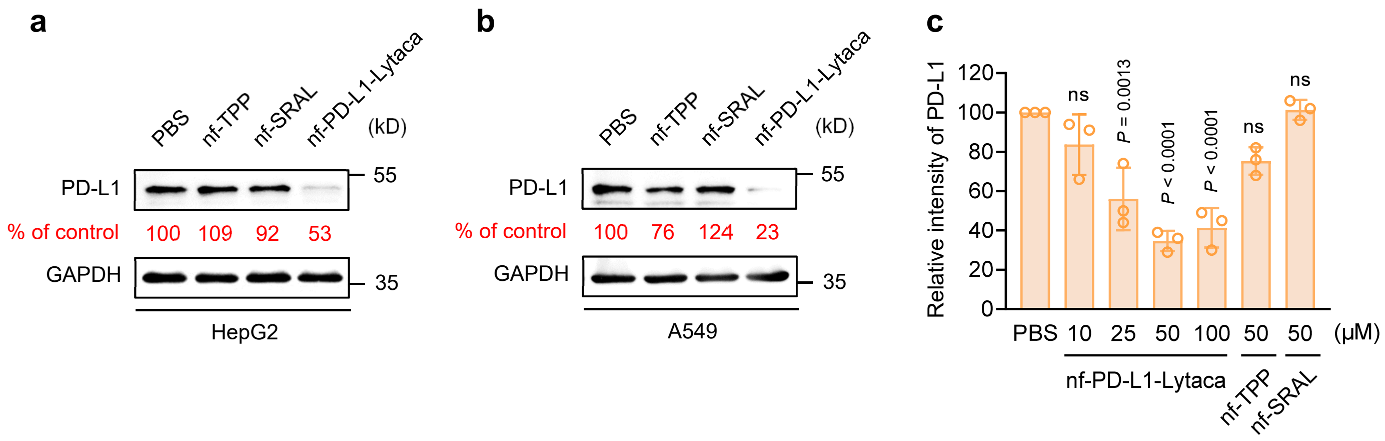

Supplementary Fig. 18 nf-PD-L1-Lytaca enable degradation of the membrane protein PD-L1. a) Western blot of PD-L1 in HepG2 cells treated with 50 μM nf-TPP, nf-SRAL or nf-PD-L1-Lytaca for 12 h. **b)** Western blot of PD-L1 in A549 cells treated with 50 μM nf-TPP, nf-SRAL or nf-PD-L1-Lytaca for 12 h. Densitometry was used to calculate protein levels, and data were normalized to the PBS control. **c)** Western blot analysis of membrane PD-L1 in RAW264.7 cells treated with 10, 25, 50 and 100 μM nf-PD-L1-Lytaca, 50 μM nf-TPP or nf-SRAL for 12 h. Data are presented as the mean ± SD, and error bars represent the standard deviation of biological replicates (n = 3). P values were determined by one-way ANOVA with Tukey's multiple comparisons test, ns, no significance.

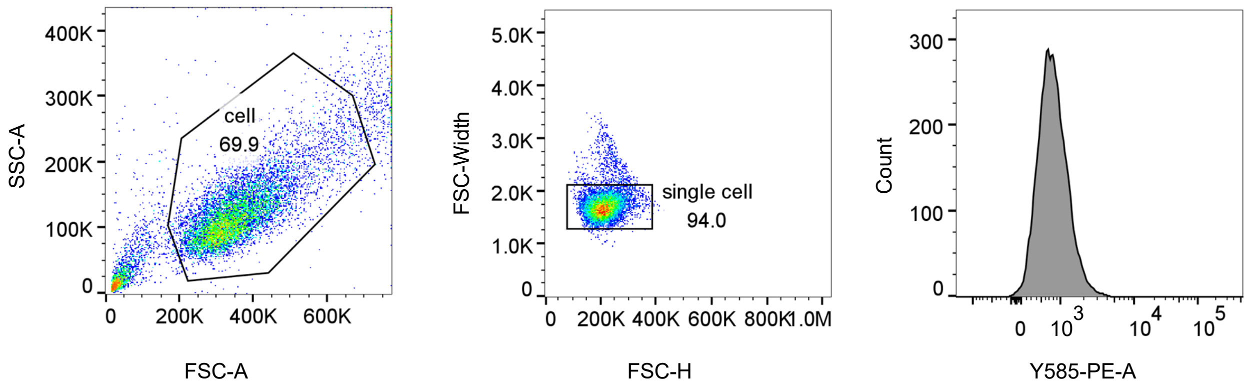

Supplementary Fig. 19 Representative flow cytometry gating for intracellular IL-17A in RAW264.7 cells. Debris was gated out by the FSC and SSC area, and single cells were gated on FSC-H and FSC-Width.

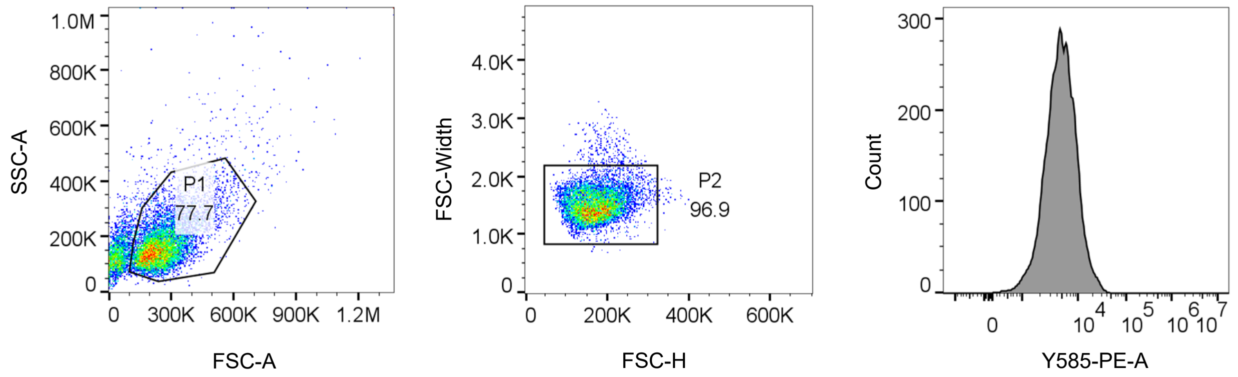

Supplementary Fig. 20 Representative flow cytometry gating for surface PD-L1 in RAW264.7 cells. Debris was gated out by the FSC and SSC area, and single cells were gated on FSC-H and FSC-Width.

Characterization of peptides

Peptide1 fi-SRAL

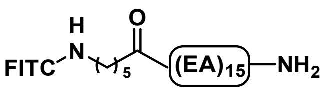

fi-SRAL

Chemical Formula: $C_{147}H_{205}N_{33}O_{66}S$

Exact Mass: 3520.34

Molecular Weight: 3522.48

Upon deprotection of the side chain, the crude peptide was dissolved in 16 ml of acetonitrile/water (50/50, v/v) solution and purified by reverse high performance liquid chromatography (RP-HPLC) (linear concentration gradient of 25-40% for mobile phase B, using an Exsil Pure 300 C18 column). The retention times of the products were 9.50-11.30 min. The product fraction was collected and lyophilized under reduced pressure to give peptide fi-SRAL (12.6 mg, 7.2% total yield) as a fluffy yellow solid.

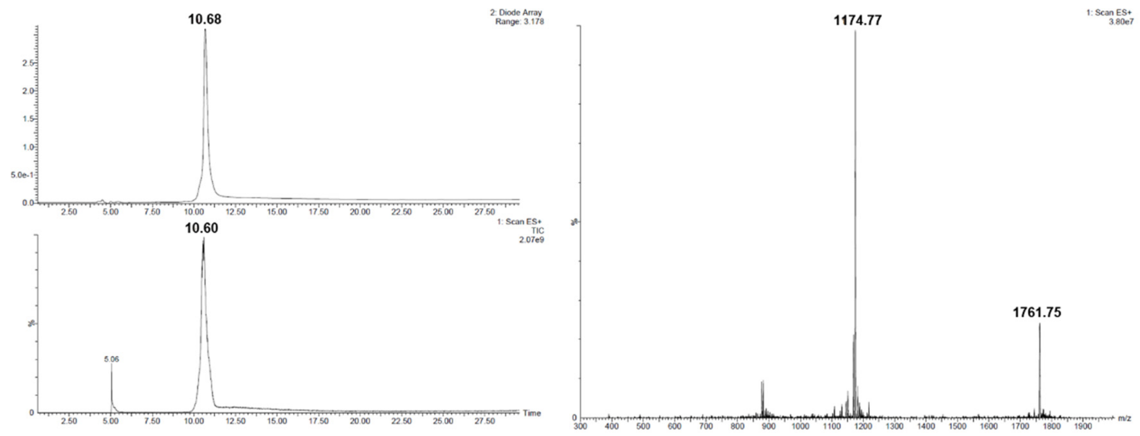

Supplementary Figure 21 HPLC-MS analysis of peptide fi-SRAL. Left: UV (Top) and MS (Down) traces (Liner gradient of 25-40% solvent B over 30 min, Agilent C18 column, t_R = 10.68 min); Right: ESI-MS$^+$ data. Calcd mass for $C_{147}H_{205}N_{33}O_{66}S$: 3520.34 Da (average isotopes), $[M+2H]^{2+}$ m/z = 1761.17, $[M+3H]^{3+}$ m/z = 1174.46; observed: 1761.75, 1174.77.

Peptide2 fi-17Abp

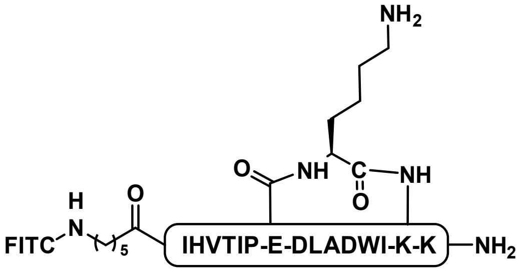

fi-17Abp

Chemical Formula: $C_{116}H_{165}N_{25}O_{28}S$
Exact Mass: 2388.20
Molecular Weight: 2389.80

Upon deprotection of the side chain, the crude peptide was dissolved in 16 ml of acetonitrile/water (50/50, v/v) solution and purified by RP-HPLC (linear concentration gradient of 30-55% for mobile phase B, using an Exsil Pure 300 C18 column). The retention times of the products were 25.00-26.70 min. The product fraction was collected and lyophilized under reduced pressure to give Peptide fi-17Abp (12.6 mg, 6.5% total yield) as a fluffy yellow solid.

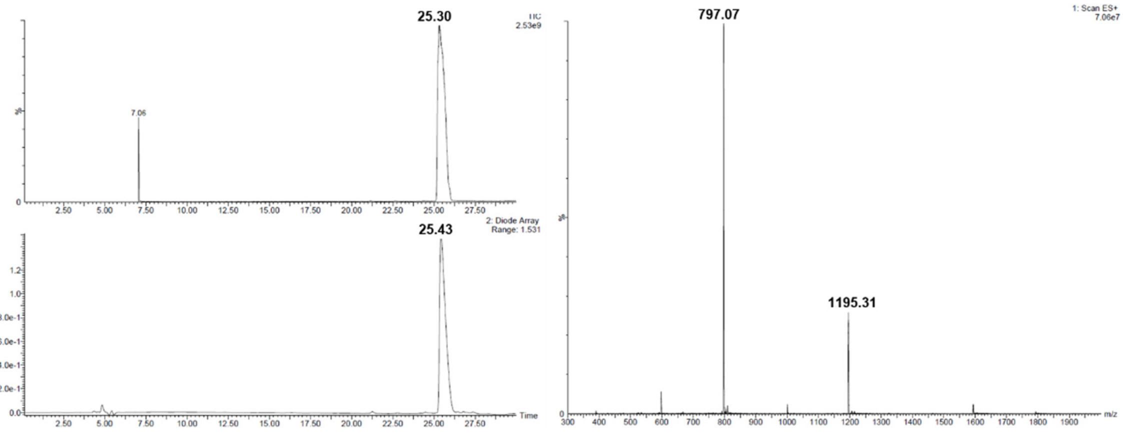

Supplementary Figure 22 HPLC-MS analysis of peptide fi-17Abp. Left: MS (Top) and UV (Down) (Liner gradient of 30-55% solvent B over 30 min, Agilent C18 column, t_R = 25.43 min); Right: ESI-MS^+ data. Calcd mass for $C_{116}H_{165}N_{25}O_{28}S$: 2388.20 Da (average isotopes), $[M+2H]^{2+}$ *m/z* = 1195.10, $[M+3H]^{3+}$ *m/z* = 797.06; observed: 1195.31, 797.07.

Peptide3 fi-SRAL-17Abp chimera

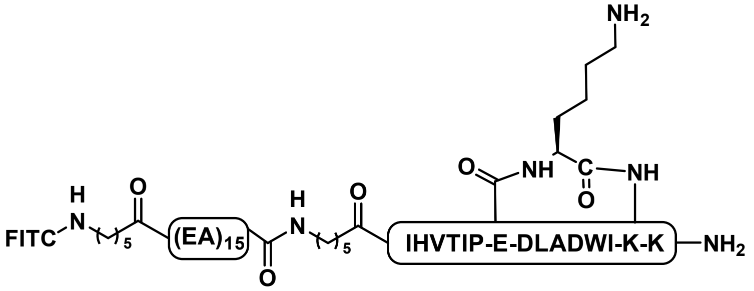

fi-SRAL-17Abp chimera
Chemical Formula: $C_{242}H_{356}N_{56}O_{89}S$
Exact Mass: 5502.48
Molecular Weight: 5505.87

Upon deprotection of the side chain, the crude peptide was dissolved in 16 ml of acetonitrile/water (50/50, v/v) solution and purified by RP-HPLC (linear concentration gradient of 30-60% for mobile phase B, using an Exsil Pure 300 C18 column). The retention times of the products were 16.90-18.30 min. The product fraction was collected and lyophilized under reduced pressure to give peptide fi-SRAL-17Abp chimera (5.3 mg, 1.9% total yield) as a fluffy yellow solid.

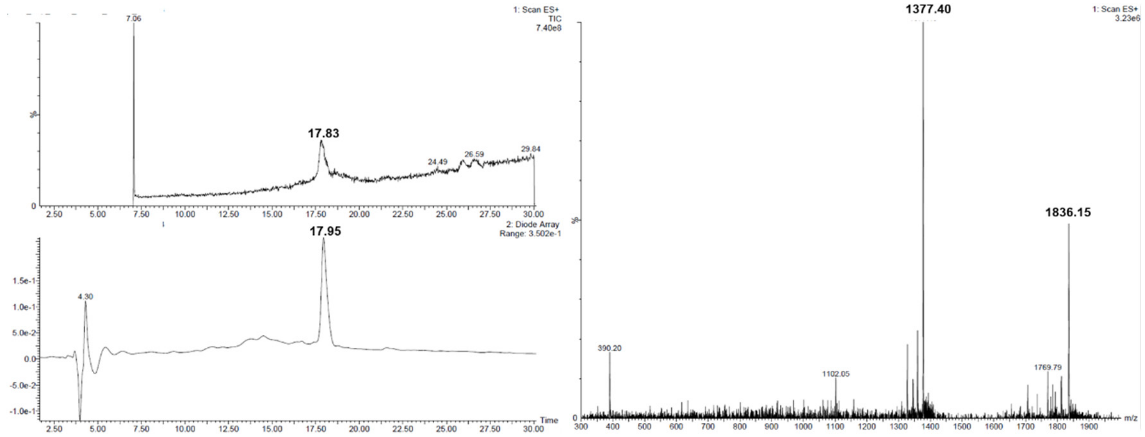

Supplementary Figure 23 HPLC-MS analysis of peptide fi-SRAL-17Abp chimera. Left: MS (Top) and UV (Down) (Liner gradient of 30-60% solvent B over 30 min, Agilent C18 column, t_R = 17.95 min); Right: ESI-MS$^+$ data. Calcd mass for $C_{242}H_{356}N_{56}O_{89}S$: 5502.48 Da (average isotopes), $[M+3H]^{3+}$ *m/z* = 1835.16, $[M+4H]^{4+}$ *m/z* = 1376.62; observed: 1836.15, 1377.40.

Peptide4 rb-SRAL

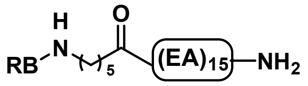

rb-SRAL
Chemical Formula: $C_{154}H_{224}N_{34}O_{63}$
Exact Mass: 3557.54
Molecular Weight: 3559.66

Upon deprotection of the side chain, the crude peptide was dissolved in 16 ml of acetonitrile/water (50/50, v/v) solution and purified by RP-HPLC (linear concentration gradient of 30-60% for mobile phase B, using an Exsil Pure 300 C18 column). The retention time of the product was 10.90-12.00 min. The product fraction was collected and lyophilized under reduced pressure to give peptide rb-SRAL (4.5mg, 6.2% total yield) as a fluffy pink solid.

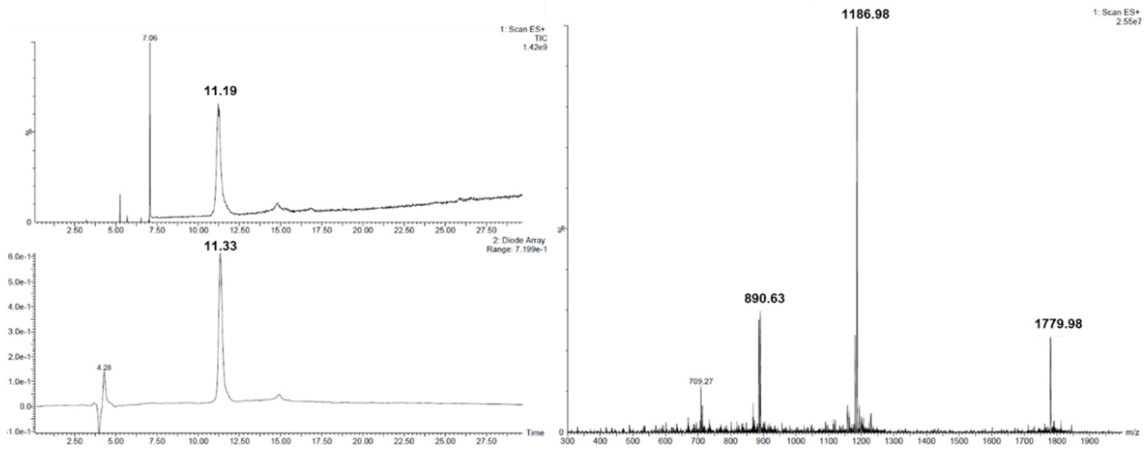

Supplementary Figure 24 HPLC-MS analysis of peptide rb-SRAL. Left: MS (Top) and UV (Down) (Liner gradient of 30-60% solvent B over 30 min, Agilent C18 column, t_R = 11.33 min); Right: ESI-MS$^+$ data. Calcd mass for $C_{154}H_{224}N_{34}O_{63}$: 3557.54 Da (average isotopes), $[M+2H]^{2+}$ *m/z* = 1779.77, $[M+3H]^{3+}$ *m/z* = 1186.85, $[M+4H]^{4+}$ *m/z* = 890.39; observed: 1779.98, 1186.98, 890.63.

Peptide5 fm-SRAL

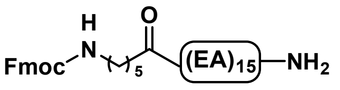

fm-SRAL

Chemical Formula: $C_{141}H_{204}N_{32}O_{63}$
Exact Mass: 3353.37
Molecular Weight: 3355.34

Upon deprotection of the side chain, the crude peptide was dissolved in 16 ml of acetonitrile/water (50/50, v/v) solution and purified by RP-HPLC (linear concentration gradient of 30-60% for mobile phase B, using an Exsil Pure 300 C18 column). The retention times of the products were 16.00 -18.60 min. The product fraction was collected and lyophilized under reduced pressure to give peptide fm-SRAL (30 mg, 8.9% total yield) as a fluffy solid.

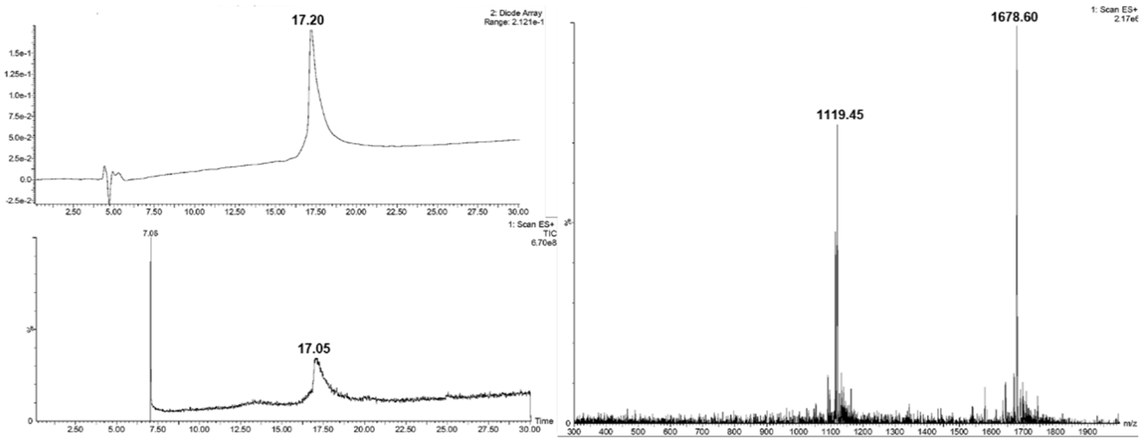

Supplementary Figure 25 HPLC-MS analysis of peptide fm-SRAL. Left: UV (Top) and MS (Down) (Liner gradient of 30-60% solvent B over 30 min, Agilent C18 column, t_R = 17.20 min); Right: ESI-MS$^+$ data. Calcd mass for $C_{141}H_{204}N_{32}O_{63}$: 3353.37 Da (average isotopes), $[M+2H]^{2+}$ *m/z* =1677.69, $[M+3H]^{3+}$ *m/z* = 1118.79; observed: 1678.60, 1119.45.

Peptide6 fm-17Abp

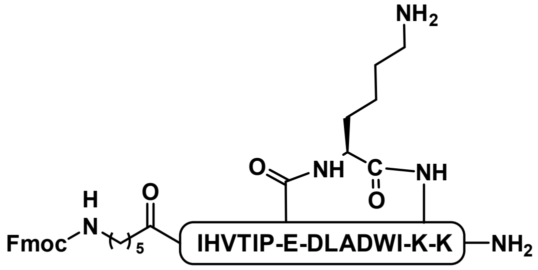

fm-17Abp

Chemical Formula: $C_{110}H_{164}N_{24}O_{25}$
Exact Mass: 2221.23
Molecular Weight: 2222.67

Upon deprotection of the side chain, the crude peptide was dissolved in 16 ml of acetonitrile/water (50/50, v/v) and purified by RP-HPLC (linear concentration gradient of 40-55% for mobile phase B, using an Exsil Pure 300 C18 column). The retention times of the products were 24.00 -25.25 min. The product fraction was collected and lyophilized under reduced pressure to give peptide fm-17Abp (15 mg, 6.8 % total yield) as a fluffy solid.

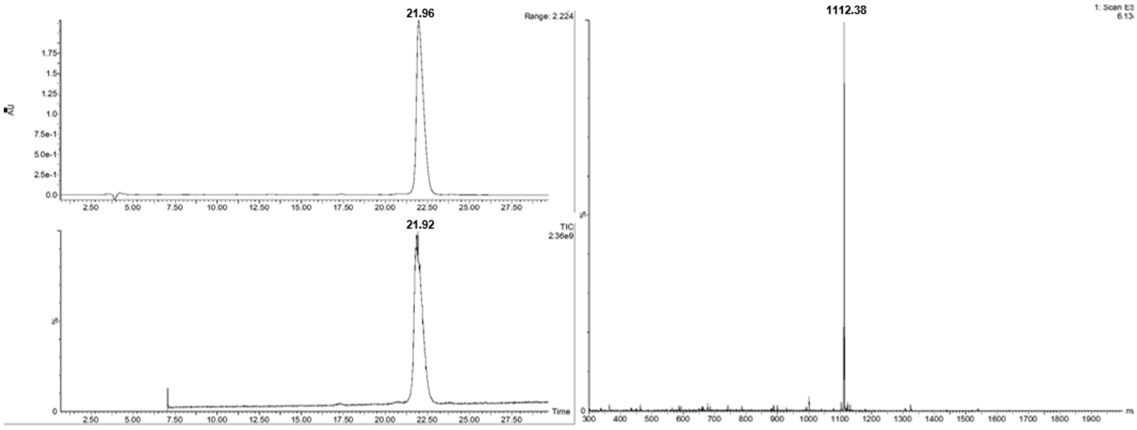

Supplementary Figure 26 HPLC-MS analysis of peptide fm-SRAL. Left: UV (Top) and MS (Down) (Liner gradient of 40-55% solvent B over 30 min, Agilent C18 column, t_R = 21.96 min); Right: ESI-MS$^+$ data. Calcd mass for $C_{110}H_{164}N_{24}O_{25}$: 2221.23 Da (average isotopes), $[M+2H]^{2+}$ *m/z* = 1111.62; observed: 1112.38.

Peptide7 ac-SRAL

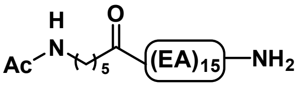

ac-SRAL

Chemical Formula: $C_{128}H_{196}N_{32}O_{62}$

Exact Mass: 3173.32

Molecular Weight: 3175.14

Upon deprotection of the side chain, the crude peptide was dissolved in 16 ml of acetonitrile/water (50/50, v/v) solution and purified by RP-HPLC (linear concentration gradient of 20-40% for mobile phase B, using an Exsil Pure 300 C18 column). The retention times of the products were 16.00 -18.00 min. The product fraction was collected and lyophilized under reduced pressure to give ac-SRAL (21 mg, total yield 6.6%) as a fluffy solid.

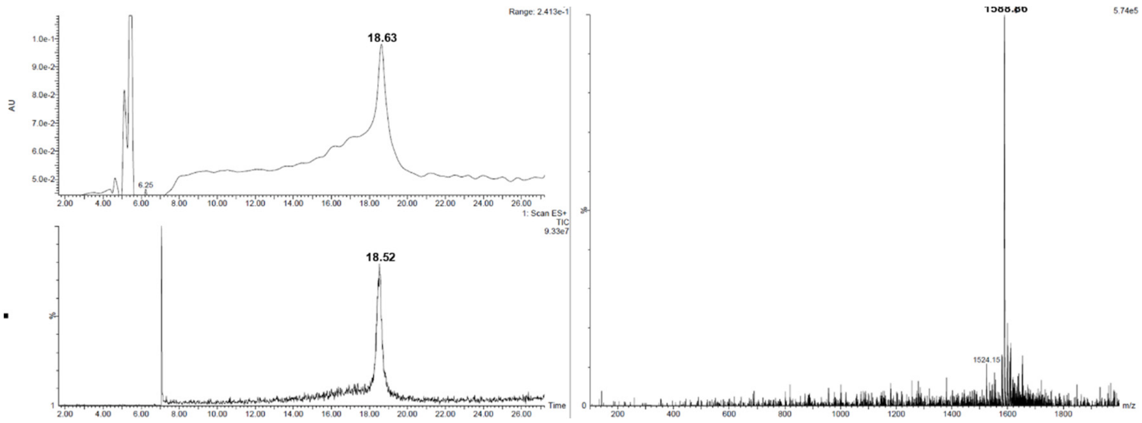

Supplementary Figure 27 HPLC-MS analysis of peptide ac-SRAL. Left: UV (Top) and MS (Down) (Liner gradient of 20-40% solvent B over 30 min, Agilent C18 column, t_R =18.63 min); Right: ESI-MS$^+$ data. Calcd mass for $C_{128}H_{196}N_{32}O_{62}$: 3173.32 Da (average isotopes), $[M+2H]^{2+}$ *m/z* =1587.66; observed: 1588.86.

Peptide8 ac-17Abp

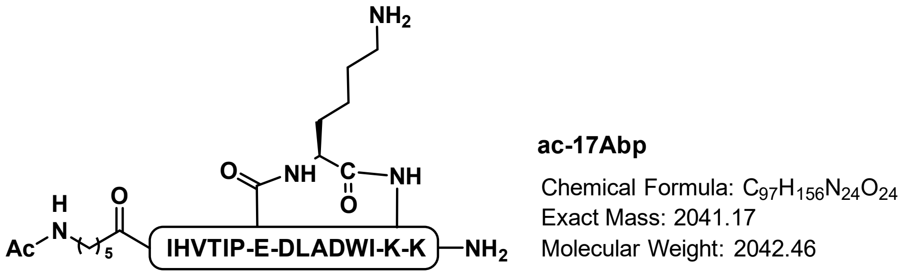

Upon deprotection of the side chain, the crude peptide was dissolved in 16 ml of acetonitrile/water (50/50, v/v) and purified by RP-HPLC (linear concentration gradient of 30-60% for mobile phase B, using an Exsil Pure 300 C18 column). The retention times of the products were 15.60 -17.00 min. The product fraction was collected and lyophilized under reduced pressure to give peptide ac-17Abp (11 mg, total yield 5.4%) as a fluffy solid.

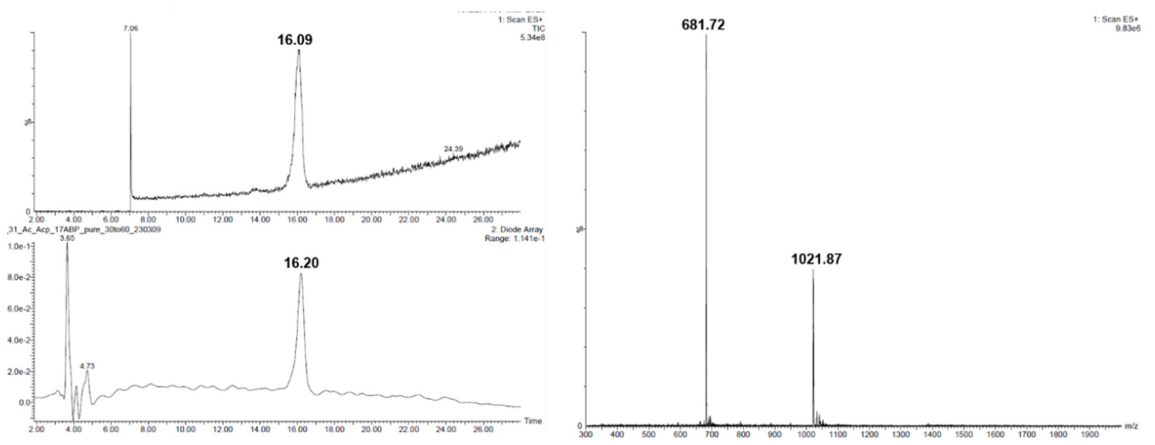

Supplementary Figure 28 HPLC-MS analysis of peptide ac-17Abp. Left: MS (Top) and UV (Down) (Liner gradient of 30-60% solvent B over 30 min, Agilent C18 column, t_R = 16.20 min); Right: ESI-MS$^+$ data. Calcd mass for $C_{97}H_{156}N_{24}O_{24}$: 2041.17 Da (average isotopes), $[M+2H]^{2+}$ *m/z* =1021.59, $[M+3H]^{3+}$ *m/z* = 681.39; observed: 1021.87, 681.72.

Peptide9 fm-TPP

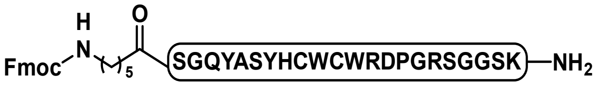

fm-TPP

Chemical Formula: $C_{128}H_{172}N_{36}O_{34}S_2$
Exact Mass: 2821.23
Molecular Weight: 2823.12

Upon deprotection of the side chain, the crude peptide was dissolved in 16 ml of acetonitrile/water (50/50, v/v) solution and purified by RP-HPLC (linear concentration gradient of 20-50% for mobile phase B, using an Exsil Pure 300 C18 column). The retention times of the products were 22.70 -24.00 min. The product fraction was collected and lyophilized under reduced pressure to give peptide fm-TPP (4.4 mg, total yield 7.8%) as a fluffy solid.

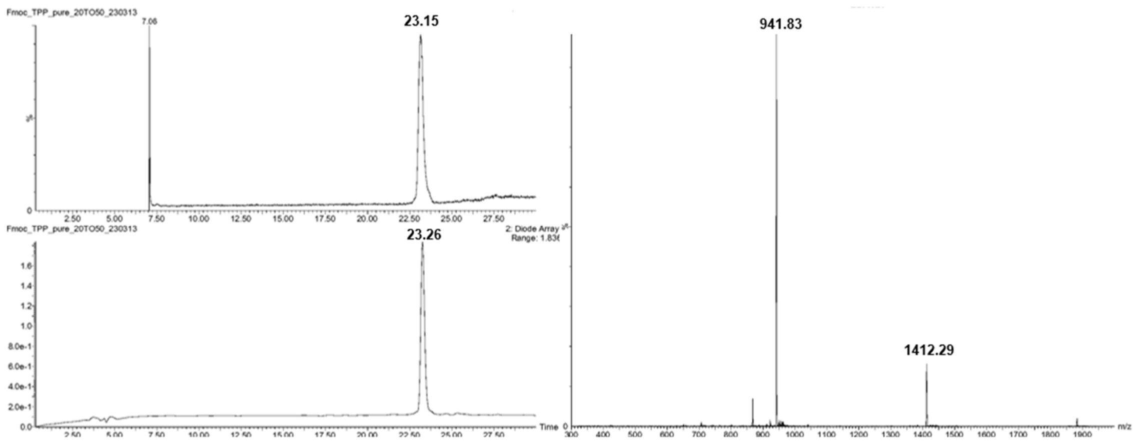

Supplementary Figure 29 HPLC-MS analysis of peptide fm-TPP. Left: MS (Top) and UV (Down) (Liner gradient of 20-50% solvent B over 30 min, Agilent C18 column, t_R = 23.26 min); Right: ESI-MS^+ data. Calcd mass for $C_{128}H_{172}N_{36}O_{34}S_2$: 2821.23 Da (average isotopes), $[M+2H]^{2+}$ *m/z* =1411.62, $[M+3H]^{3+}$ *m/z* = 941.41; observed: 1412.29, 941.83.

Peptide10 nf-TPP

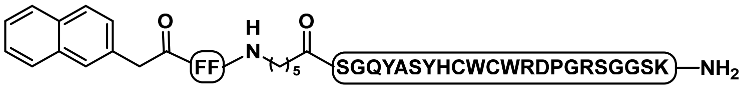

nf-TPP

Chemical Formula: $C_{143}H_{188}N_{38}O_{35}S_2$

Exact Mass: 3061.35

Molecular Weight: 3063.43

Upon deprotection of the side chain, the crude peptide was dissolved in 16 ml of acetonitrile/water (50/50, v/v) solution and purified by RP-HPLC (linear concentration gradient of 30-60% for mobile phase B, using an Exsil Pure 300 C18 column). The retention times of the products were 15.50 -17.50 min. The product fraction was collected and lyophilized under reduced pressure to give peptide nf-TPP (10.8 mg, total yield 8.1%) as a fluffy solid.

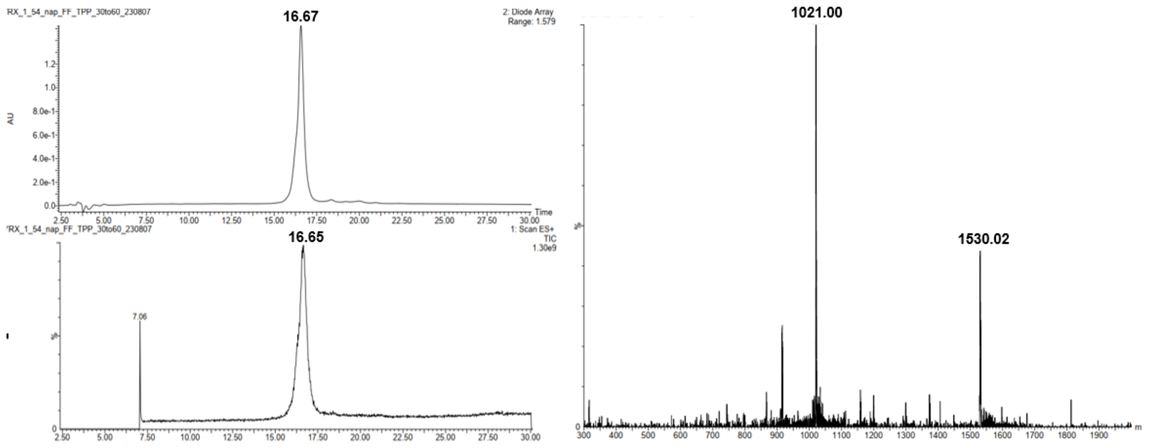

Supplementary Figure 30 HPLC-MS analysis of peptide nf-TPP. Left: UV (Top) and MS (Down) (Liner gradient of 30-60% solvent B over 30 min, Agilent C18 column, t_R = 16.67 min); Right: ESI-MS$^+$ data. Calcd mass for $C_{143}H_{188}N_{38}O_{34}S_2$: 3061.35 Da (average isotopes), $[M+2H]^{2+}$ *m/z* =1531.68, $[M+3H]^{3+}$ *m/z* = 1021.45; observed: 1530.02, 1021.00.

Peptide11 nf-SRAL

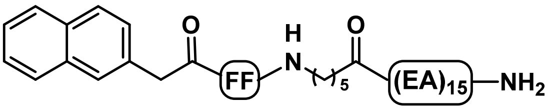

nf-SRAL

Chemical Formula: $C_{156}H_{220}N_{34}O_{64}$

Exact Mass: 3593.50

Molecular Weight: 3595.65

Upon deprotection of the side chain, the crude peptide was dissolved in 16 ml of acetonitrile/water (50/50, v/v) solution and purified by RP-HPLC (linear concentration gradient of 25-55% for mobile phase B, using an Exsil Pure 300 C18 column). The retention times of the products were 22.80 -24.00 min. The product fraction was collected and lyophilized under reduced pressure to give peptide nf-SRAL (8.8 mg, total yield 7.1%) as a fluffy solid.

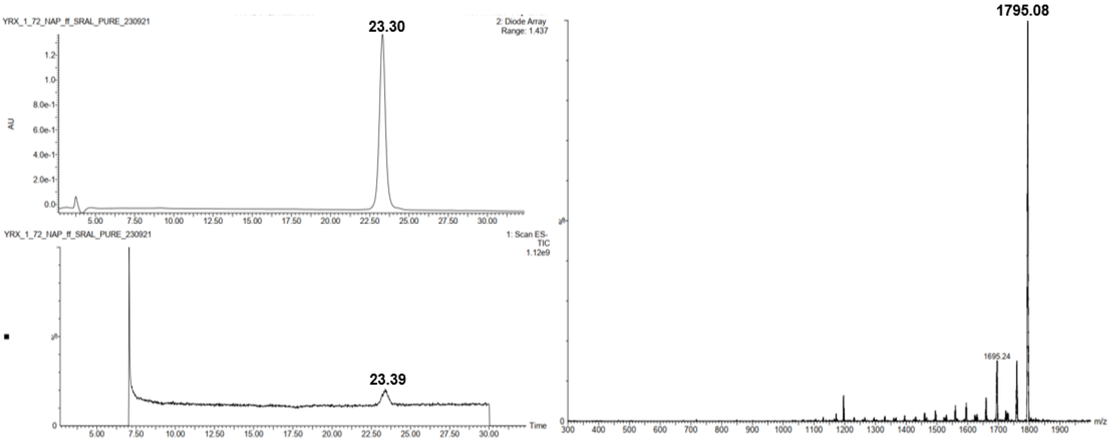

Supplementary Figure 31 HPLC-MS analysis of peptide nf-SRAL. Left: UV (Top) and MS (Down) (Liner gradient of 25-55% solvent B over 30 min, Agilent C18 column, t_R = 23.30 min); Right: ESI-MS$^-$ data. Calcd mass for $C_{156}H_{220}N_{34}O_{64}$: 3593.50 Da (average isotopes), $[M-2H]^{2-}$ *m/z* =1795.75; observed: 1795.08.

Peptide12 fm-nbp

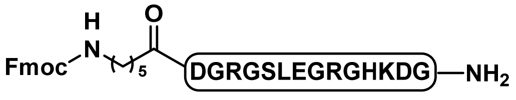

fm-nbp

Chemical Formula: $C_{77}H_{115}N_{25}O_{24}$
Exact Mass: 1773.85
Molecular Weight: 1774.92

Upon deprotection of the side chain, the crude peptide was dissolved in 16 ml of acetonitrile/water (50/50, v/v) solution and purified by RP-HPLC (linear concentration gradient of 20-45% for mobile phase B, using an Exsil Pure 300 C18 column). The retention times of the products were 16.00 -18.00 min. The product fraction was collected and lyophilized under reduced pressure to give peptide fm-nbp (15.6 mg, total yield 7.9%) as a fluffy solid.

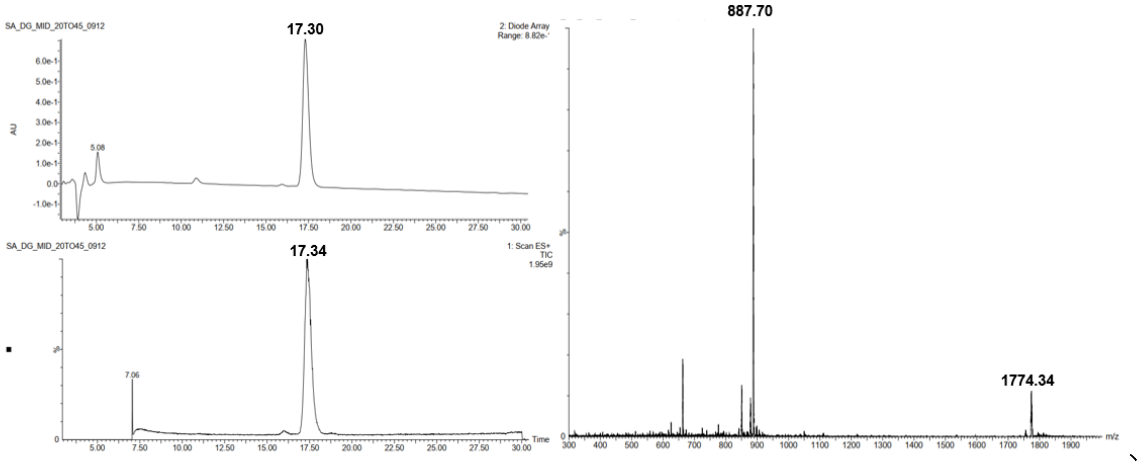

Supplementary Figure 32 HPLC-MS analysis of peptide fm-control peptide. Left: UV (Top) and MS (Down) (Liner gradient of 25-45% solvent B over 30 min, Agilent C18 column, t_R = 17.30 min); Right: ESI-MS$^+$ data. Calcd mass for $C_{77}H_{115}N_{25}O_{24}$: 1773.85 Da (average isotopes), $[M+H]^+$ *m/z* =1774.85, $[M+2H]^{2+}$ *m/z* =887.93; observed: 1774.34,887.70.

Peptide13 fm-SRAL-acp-17Abp

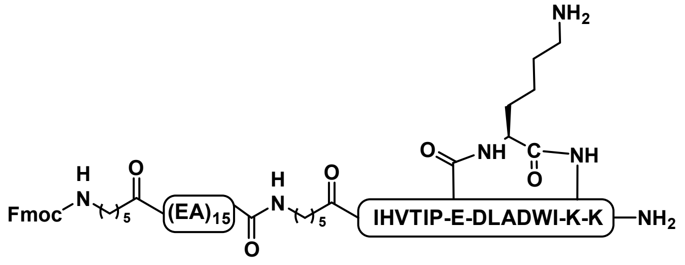

fm-SRAL-Acp-17Abp
Chemical Formula: $C_{236}H_{355}N_{55}O_{86}$
Exact Mass: 5335.51
Molecular Weight: 5338.74

Upon deprotection of the side chain, the crude peptide was dissolved in 16 ml of acetonitrile/water (50/50, v/v) solution and purified by RP-HPLC (linear concentration gradient of 30-60% for mobile phase B, using an Exsil Pure 300 C18 column). The retention times of the products were 20.60-22.00 min. The product fraction was collected and lyophilized under reduced pressure to give peptide fm-SRAL-acp-17Abp (11.6 mg, total yield 9.9%) as a fluffy solid.

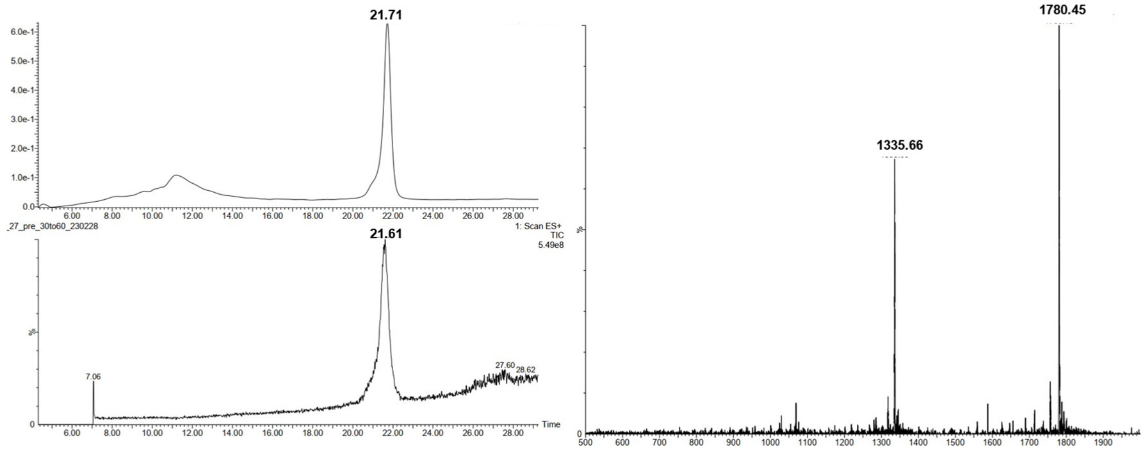

Supplementary Figure 33 HPLC-MS analysis of peptide fm-SRAL-acp-17Abp. Left: UV (Top) and MS (Down) (Liner gradient of 25-45% solvent B over 30 min, Agilent C18 column, t_R = 21.71 min); Right: ESI-MS$^+$ data. Calcd mass for $C_{236}H_{355}N_{55}O_{86}$: 5335.51 Da (average isotopes), [M+3H]3$^+$ *m/z* =1779.50, [M+4H]$^{4+}$ *m/z* =1334.88; observed: 1780.45,1335.66.

Peptide14 fm-SRAL-Anc-17Abp

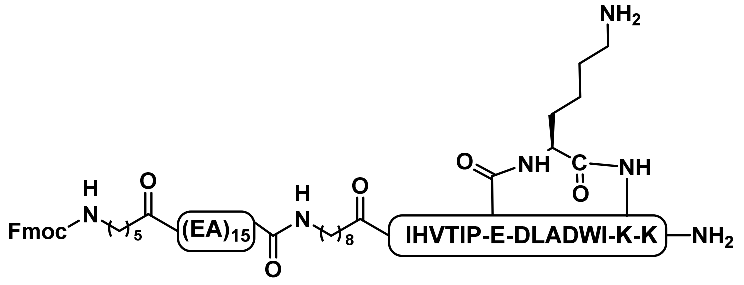

Upon deprotection of the side chain, the crude peptide was dissolved in 16 ml of acetonitrile/water (50/50, v/v) solution and purified by RP-HPLC (linear concentration gradient of 10-60% for mobile phase B, using an Ultimate® XB-C4 column). The retention times of the products were 24.00 -26.00 min. The product fraction was collected and lyophilized under reduced pressure to give peptide fm-SRAL-Anc-17Abp (2.6 mg, total yield 7.9%) as a fluffy solid.

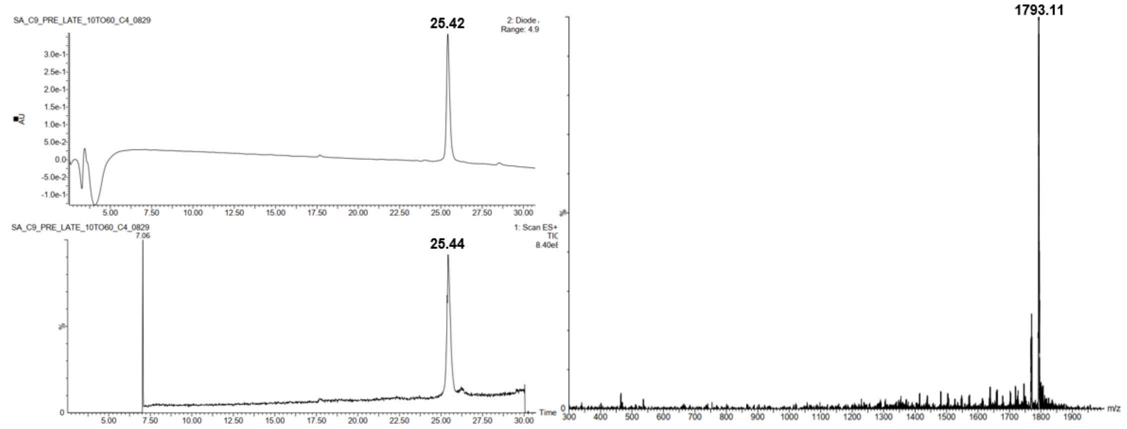

Supplementary Figure 34 HPLC-MS analysis of peptide fm-SRAL-Anc-17Abp. Left: UV (Top) and MS (Down) (Liner gradient of 10-60% solvent B over 30 min, Ultimate® XB-C4 column, t_R = 25.42 min); Right: ESI-MS$^+$ data. Calcd mass for $C_{239}H_{361}N_{55}O_{86}$: 5377.56 Da (average isotopes), $[M+3H]^{3+}$ *m/z* =1793.52; observed: 1793.11.

Peptide15 fm-SRAL-Ado-17Abp

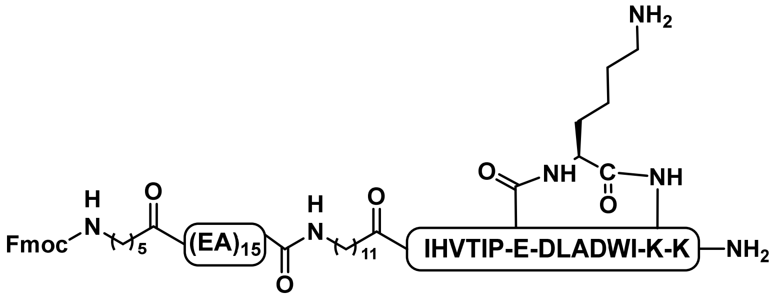

fm-SRAL-Ado-17Abp
Chemical Formula: $C_{242}H_{367}N_{55}O_{86}$
Exact Mass: 5419.60
Molecular Weight: 5422.90

Upon deprotection of the side chain, the crude peptide was dissolved in 16 ml of acetonitrile/water (50/50, v/v) solution and purified by RP-HPLC (linear concentration gradient of 20-55% for mobile phase B, using an Ultimate® XB-C4 column). The retention times of the products were 15.50 -17.00 min. The product fraction was collected and lyophilized under reduced pressure to give peptide fm-SRAL-Ado-17Abp (3.1 mg, total yield 6.3%) as a fluffy solid.

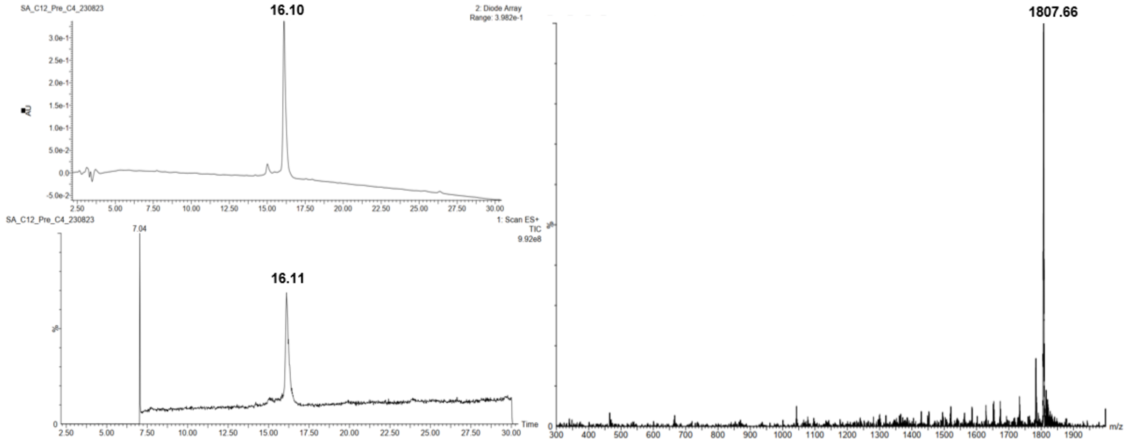

Supplementary Figure 35 HPLC-MS analysis of peptide fm-SRAL-Ado-17Abp. Left: UV (Top) and MS (Down) (Liner gradient of 20-55% solvent B over 30 min, Ultimate® XB-C4 column, t_R = 16.10 min); Right: ESI-MS$^+$ data. Calcd mass for $C_{242}H_{367}N_{55}O_{86}$: 5419.60 Da (average isotopes), $[M+3H]^{3+}$ *m/z* =1807.53; observed: 1807.66.

Peptide16 Cy3-fm-SRAL

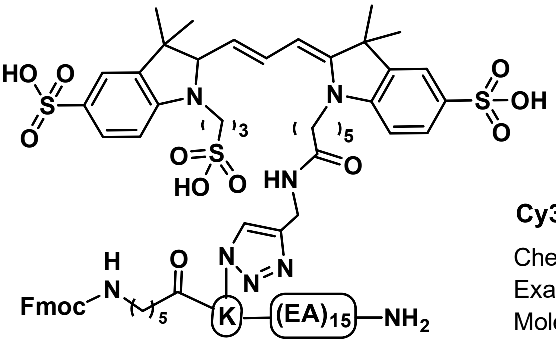

Cy3-fm-SRAL
Chemical Formula: $C_{182}H_{259}N_{39}O_{74}S_{3}$
Exact Mass: 4270.69
Molecular Weight: 4273.45

Upon deprotection of the side chain, the crude peptide was dissolved in 16 ml of acetonitrile/water (50/50, v/v) solution and purified by RP-HPLC (linear concentration gradient of 20-45% for mobile phase B, using an Ultimate® XB-C4 column). The retention times of the products were 16.00 -17.50 min. The product fraction was collected and lyophilized under reduced pressure to give peptide Cy3-fm-SRAL (5.1 mg, total yield 4.3%) as a fluffy red solid.

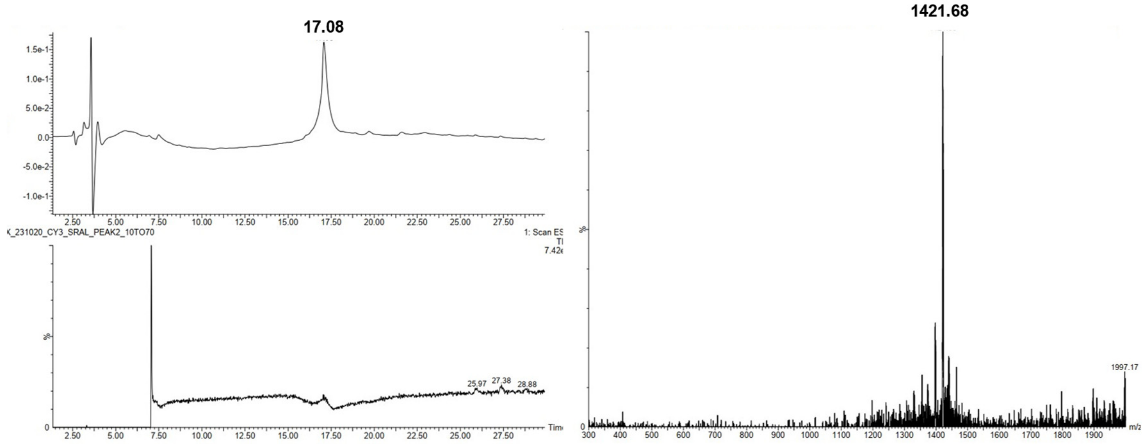

Supplementary Figure 36 HPLC-MS analysis of peptide Cy3-fm- SRAL. Left: UV (Top) and MS (Down) (Liner gradient of 20-45% solvent B over 30 min, Ultimate® XB-C4 column, t_R = 17.08 min); Right: ESI-MS$^-$ data. Calcd mass for $C_{182}H_{259}N_{39}O_{74}S_{3}$: 4270.69 Da (average isotopes), $[M-3H]^{3-}$ *m/z* =1422.56; observed: 1421.68.

NMR Spectra of Glycosylated Substrates

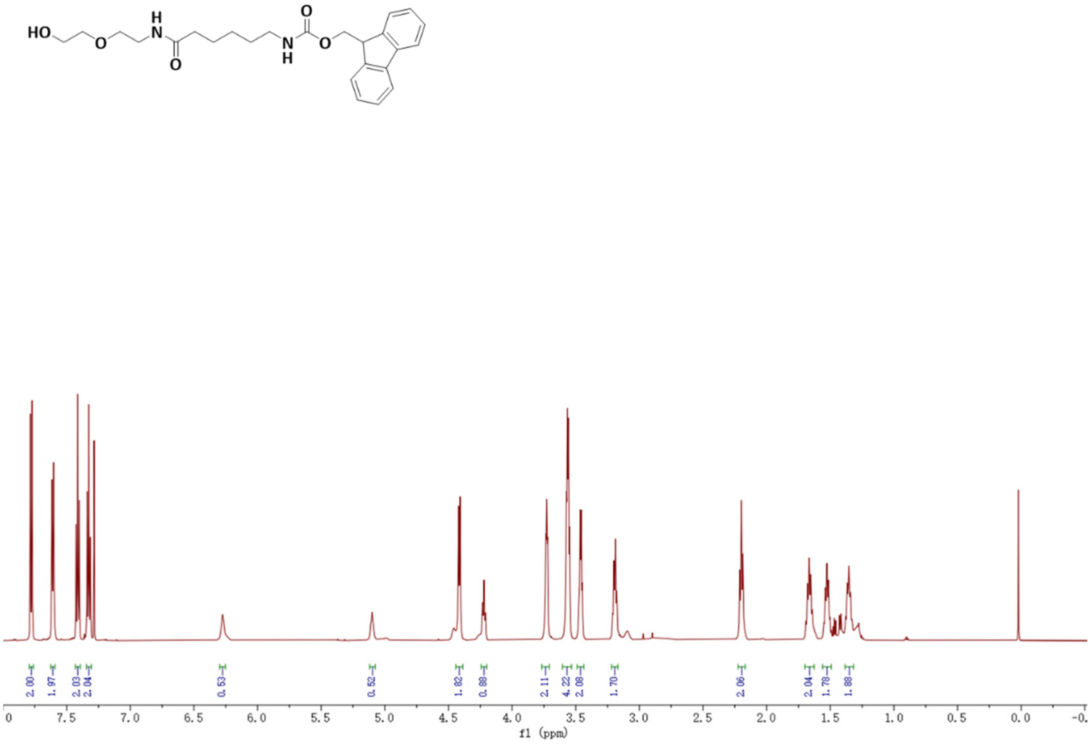

Supplementary Figure 37 Compound S2 -^{1}H NMR Spectrum - $CDCl_3$, 600 MHz

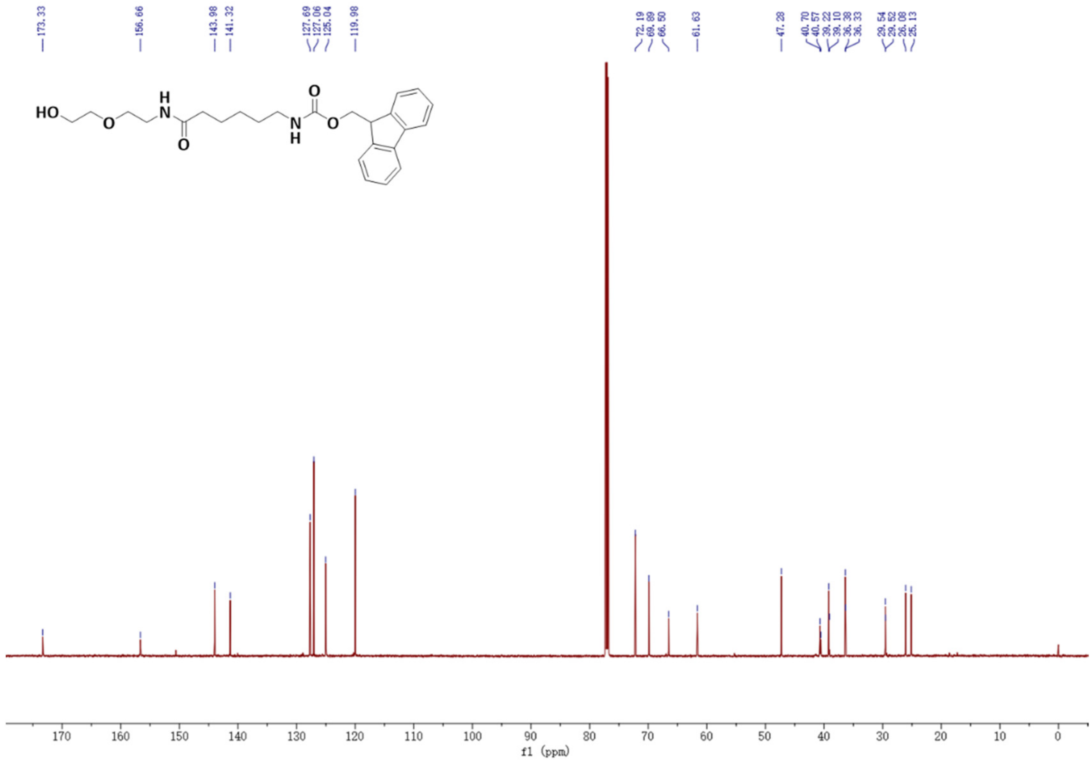

Supplementary Figure 38 Compound S2 -^{13}C NMR Spectrum - $CDCl_3$, 151 MHz

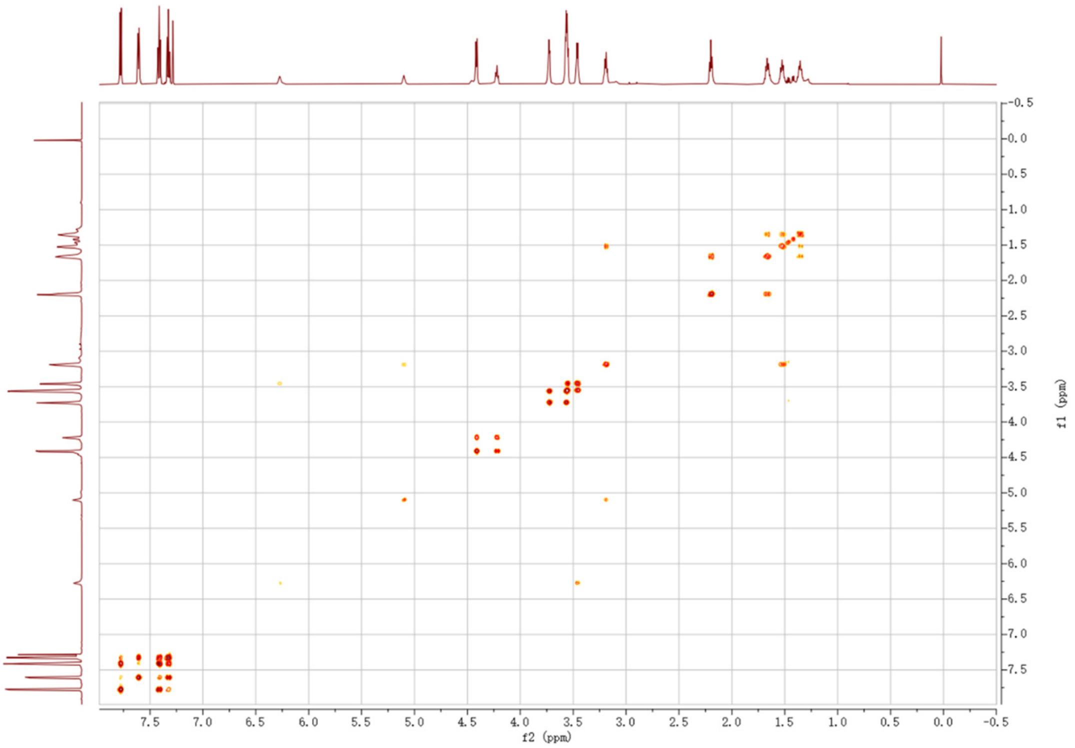

Supplementary Figure 39 Compound S2 -HH-COSY NMR Spectrum - $CDCl_3$, 600 MHz

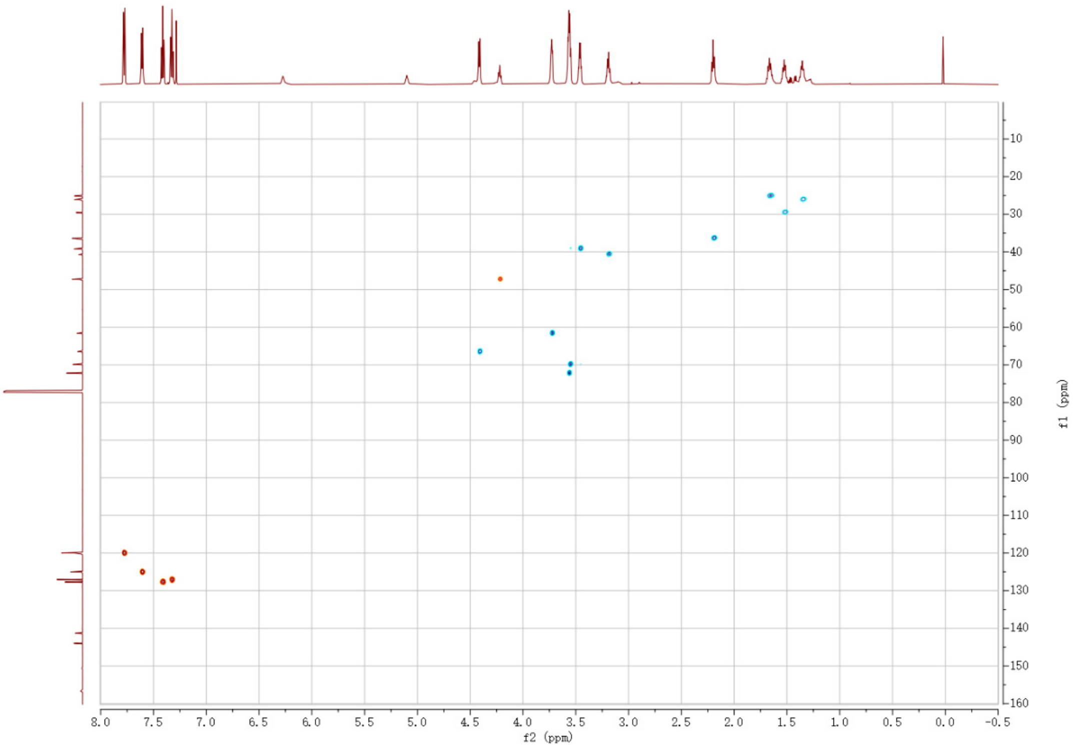

Supplementary Figure 40 Compound S2 -HSQC NMR Spectrum - $CDCl_3$, 600 MHz

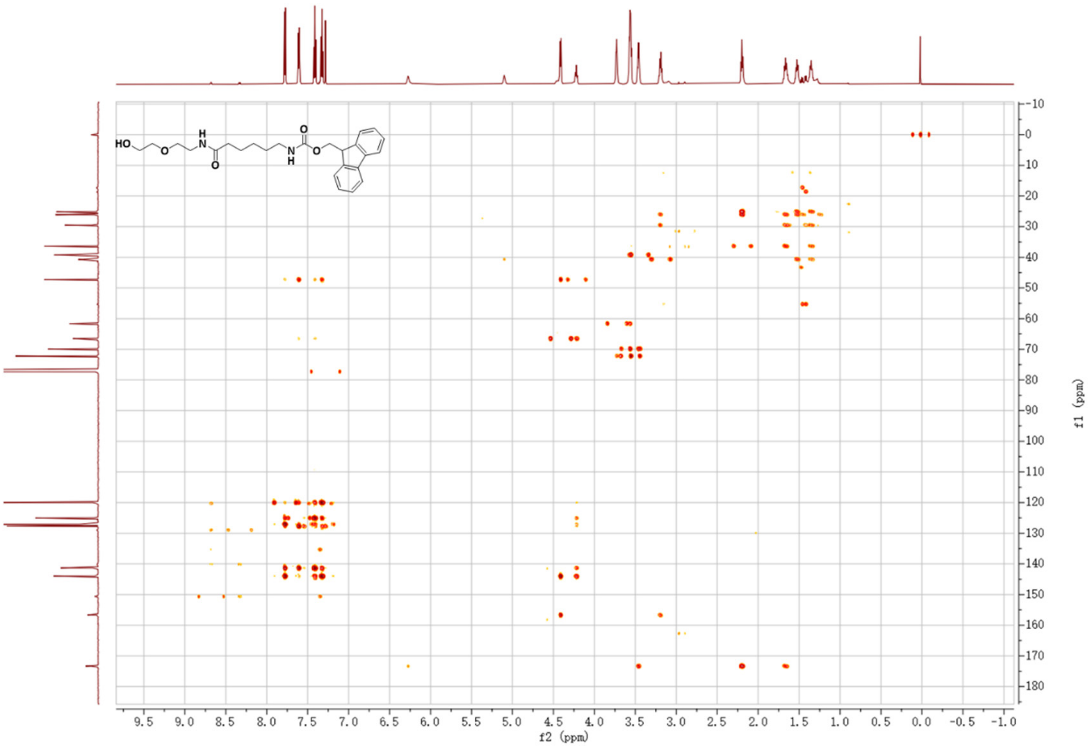

Supplementary Figure 41 Compound S2 -HMBC NMR Spectrum - $CDCl_3$, 600 MHz

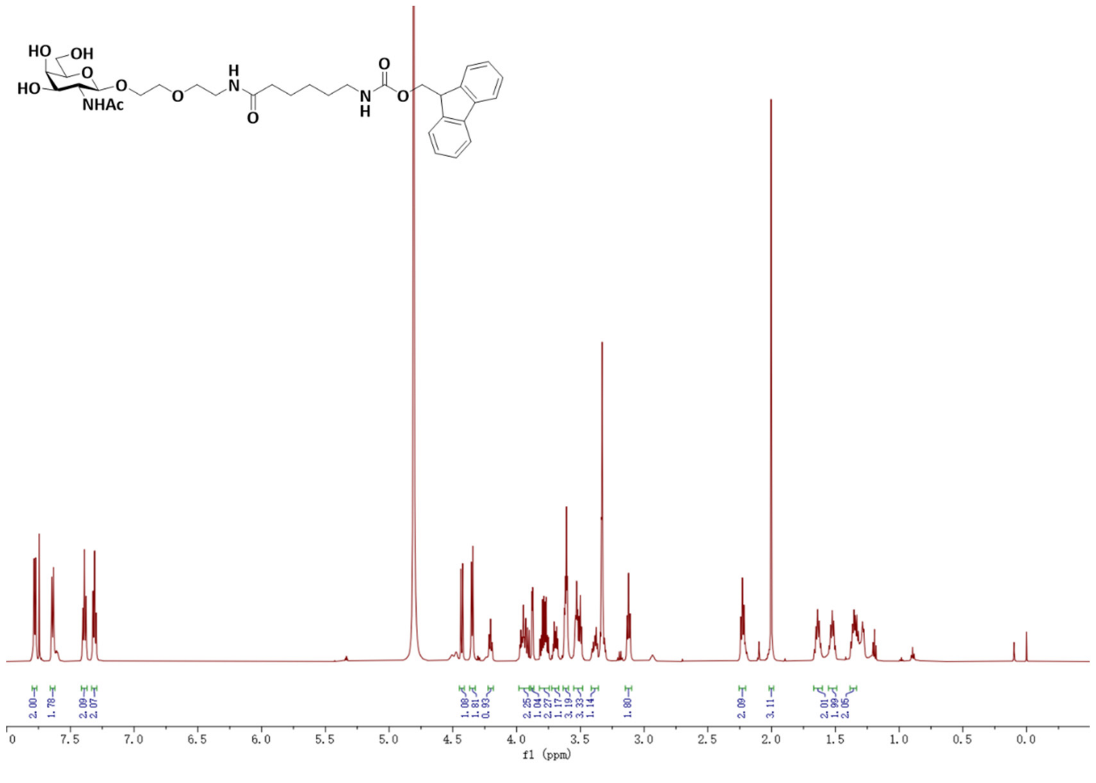

Supplementary Figure 42 Compound S5 -^{1}H NMR Spectrum - CD_3OD, 600 MHz

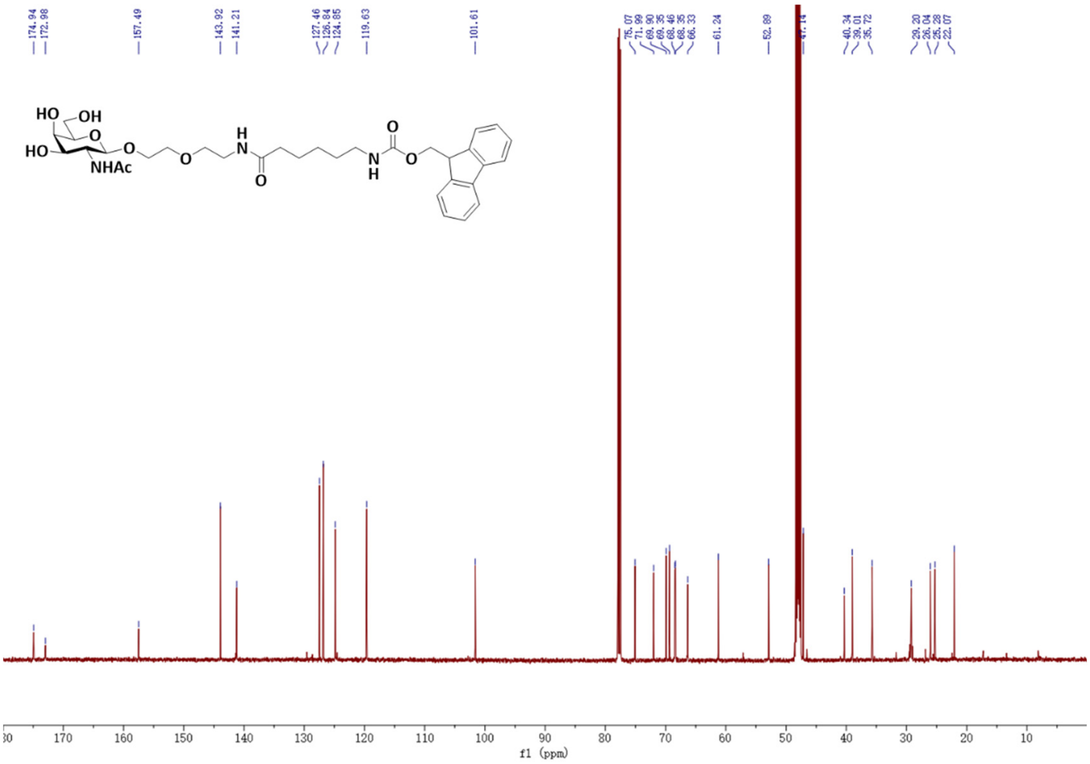

Supplementary Figure 43 Compound S5 -^{13}C NMR Spectrum - CD$_3$OD, 151 MHz

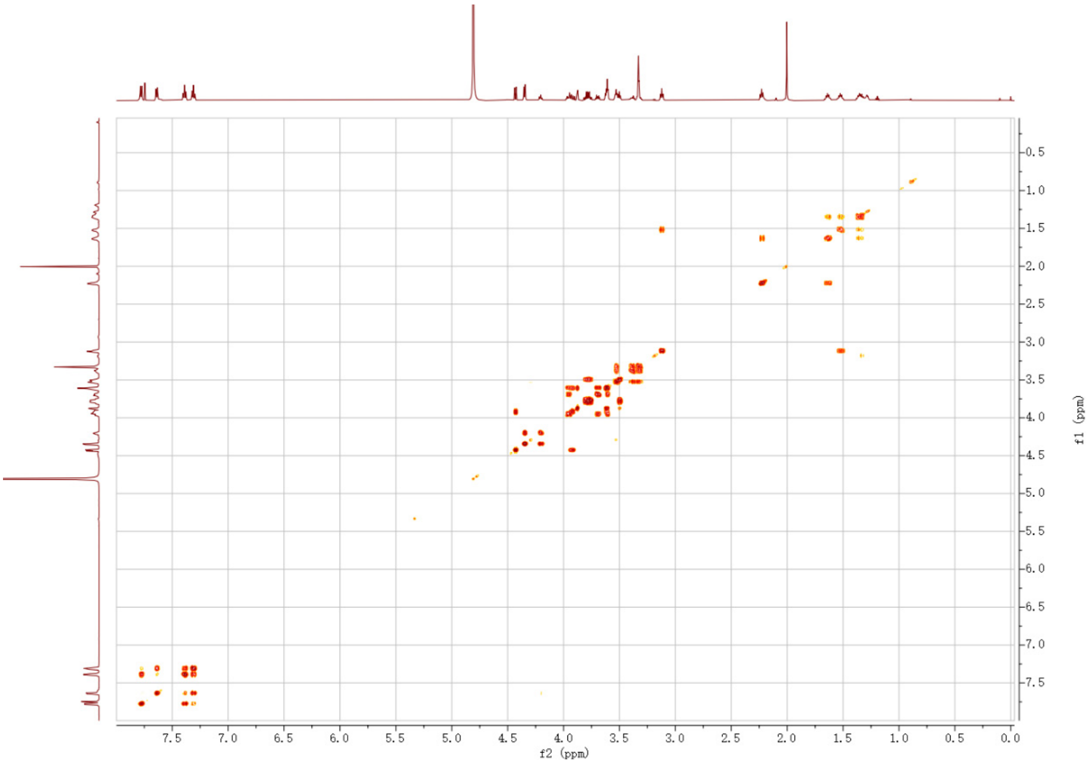

Supplementary Figure 44 Compound S5 -HH-COSY NMR Spectrum - CD$_3$OD, 600 MHz

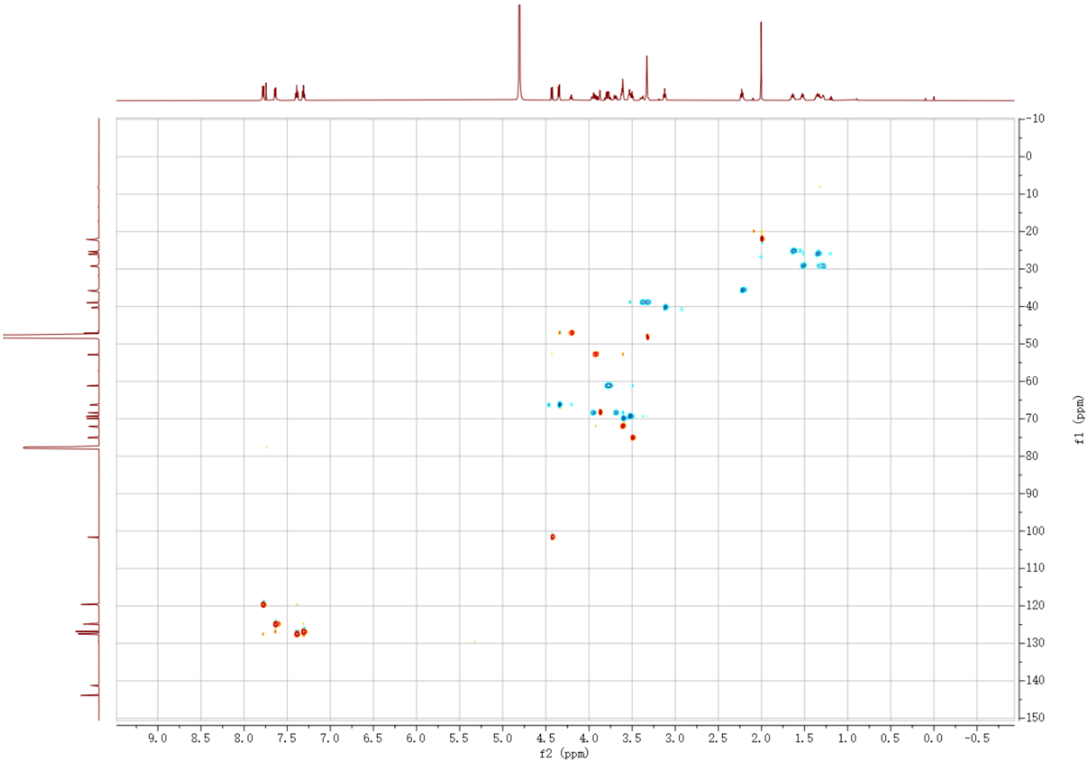

Supplementary Figure 45 Compound S5 -HSQC NMR Spectrum - CD_3OD, 600 MHz

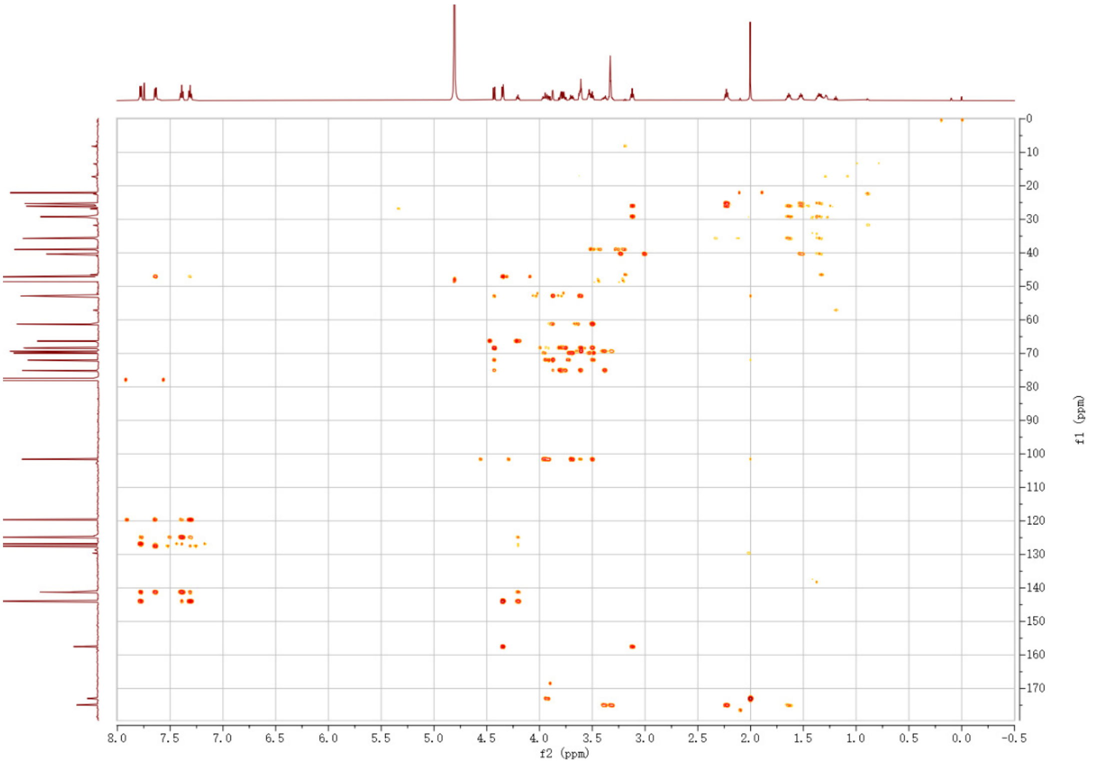

Supplementary Figure 46 Compound S5 -HMBC NMR Spectrum - CD_3OD, 600 MHz

Supplementary Tables

Primer name	Sequences (5'-3' orientation)
**gRNA scaffold**	GTTTTAGAGCTAGAAATAGCAAGTTAAAATAAGGCT AGTCCGTTATCAACTTGAAAAAGTGGCACCGAGTC GGTGCTTTT
**gRNA primer**	**Forward:** CCTCTAATACGACTCACTATAGGTGAACG TGCGTCAAATTTCAGTTTTAGAGCTAGAAATAGC **Reverse:** AAAAGCACCGACTCGGTGCC

Supplemental Table 1 Sequences of gRNA scaffold and primers for generation of SR-A knockout-cell line

Gene	Primer sequences (5'-3' orientation)
**IL-6**	**Forward:** TAGTCCTTCCTACCCCAATTTCC
	**Reverse:** TTGGTCCTTAGCCACTCCTTC
**CCL20**	**Forward:** GCCTCTCGTACATACAGACGC
	**Reverse:** CCAGTTCTGCTTTGGATCAGC
**IL-22**	**Forward:** GGTGACGACCAGAACATCCA
	**Reverse:** CAGCAGGTCCAGTTCCCCAAT
**IL-17A**	**Forward:** TTTAACTCCCTTGGCGCAAAA
	**Reverse:** CTTTCCCTCCGCATTGACAC

Supplemental Table 2 Primer sequences used for the qPCR analysis

Supplementary Methods

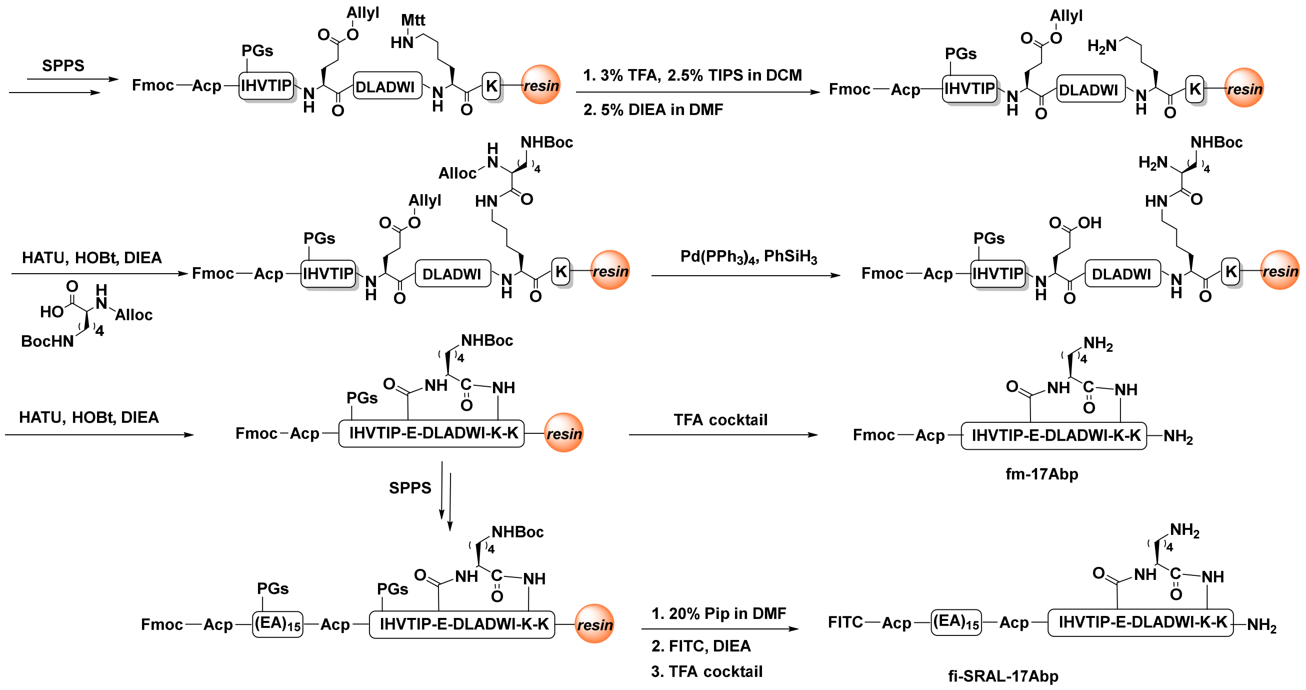

Synthesis of fi-SRAL-17Abp chimera.

After successful synthesis of the peptides, the Mtt protecting group of Lys14 was removed under 3% trifluoroacetic acid (TFA) and 2.5% triisopropylsilane(TIPS) in DCM within 5min, and repeated the procedure twice again. 5% *N,N*-Diisopropylethylamine(DIEA) in DMF for neutralization. Then, attached the *N*-Alloc-amino acid linkers to the exposed amino group via amidation. The Pd $(PPh_3)_4$ was used to remove the allyloxycarbonyl and allyloxy groups at the same time. The staple loop was finally closed via the amidation reaction between the unprotected amino and hydroxyl group.

After the next successful synthesis of the peptides, 20% of piperidine in DMF is used to remove Fmoc group from the N-terminal. Fluorescein Isothiocyanate (FITC) and DIEA in DMF for N-terminal modifications. After global deprotection, the crude peptide was dissolved in 28 mL of CH_3CN/H_2O (50/50, v/v) and further purified using RP-HPLC. The fractions were collected and lyophilized to provide peptides pure products as a fluffy yellow solid.

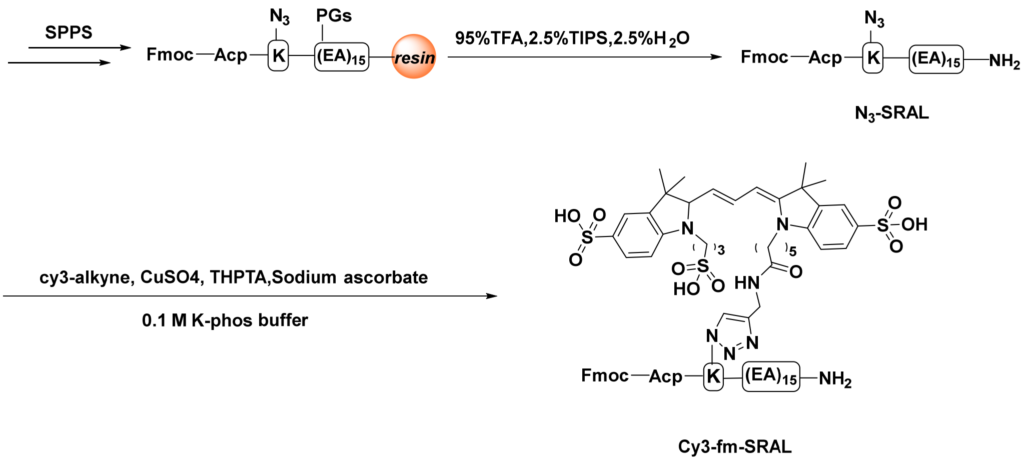

Synthesis of Cy3 labeled -fm-SRAL.

Peptides were prepared according to automated solid-phase peptide synthesis. After global deprotection and further purified using RP-HPLC, the purified N_3-SRAL peptide was dissolved to 0.5mM by potassium dihydrogen phosphate buffer. Mixed 1 mM Cy3-alkyne, 1 mM $CuSO_4$, 5 mM THPTA and 10 mM sodium ascorbate into the system and reacted for 4h. Then further purified the crude by RP-HPLC. The fractions were collected and lyophilized to provide peptides pure products as a fluffy blue solid.

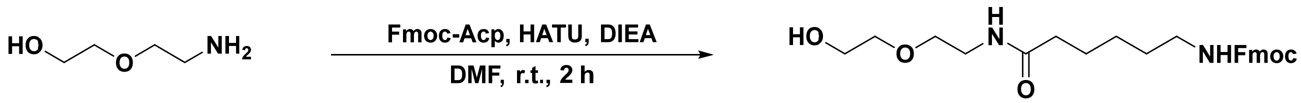

S1 S2

Synthesis of isotope labeled Mtt protected lysine(S2)

Fmoc-Acp-OH (212 mg, 0.6 mmol), HATU (210 mg, 0.55 mmol) and DIEA (187 μL, 1.1 mmol) in DMF (1 mL) was added to compound **S1** (50 μL, 0.5 mmol) in DMF (1 mL) dropwise. Then keep the mixture stirred for 2 h. After the reaction reached full conversion, the residual was extracted with EtOAc and washed with 1M HCl (x1), H_2O (x1), and brine (x1), dried over Na_2SO_4 and filtered. The filtrate was concentrated in vacuo and the resulting crude mixture was purified using silica gel column chromatography (Petroleum Ether: EtOAc = 1:1) to afford the desired product **S2** (240 mg, 91 %) as a colourless slurry. ^{1}H NMR (Chloroform-d, 600 MHz) δ 7.78 (2H, d, J = 7.6 Hz, aromatic), 7.61 (2H, d, J = 7.5 Hz, aromatic), 7.41 (2H, t, J = 7.5 Hz, aromatic), 7.33 (2H, t, J = 7.4 Hz, aromatic), 6.27 (1H, t, J = 5.7 Hz, -NH), 5.10 (1H, t, J = 6.1 Hz, -NH), 4.41 (2H, d, J = 6.9 Hz), 4.22 (1H, t, J = 6.9 Hz), 3.80-3.65 (2H, m), 3.56 (4H, q, J = 5.2 Hz), 3.46 (2H, q, J = 5.2 Hz), 3.19 (2H, q, J = 6.6 Hz), 2.20 (2H, t, J = 7.4 Hz), 1.67 (2H, p, J = 7.5 Hz), 1.53 (2H, p, J = 7.2 Hz), 1.36 (2H, q, J = 8.2 Hz); ^{13}C NMR (151 MHz, CDCl3) δ 173.33, 156.66, 143.98, 141.32, 127.69, 127.06, 125.04, 119.98, 72.19, 69.89, 66.50, 61.63, 47.28, 40.70, 40.57, 39.22, 39.10, 36.38, 36.33, 29.54, 29.52, 26.08, 25.13. HRMS (ESI): *m/z* calcd for $C_{25}H_{32}N_2O_5Na^+$ $[M+Na]^+$: 463.2341; found: 463.2349.

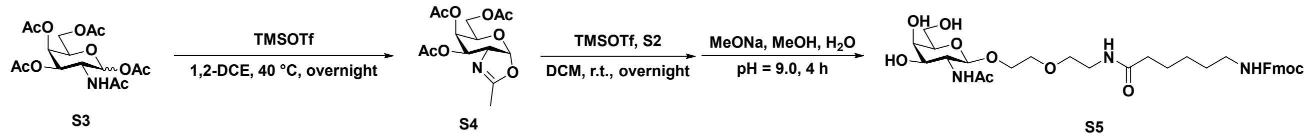

Synthesis of ASGPRL(S5)

To a solution of compound **S3** (200 mg, 0.51 mmol) in 1,2-DCE (4 mL), TMSOTf (112 μL) was added and kept it stirred at 40 °C overnight. After the reaction reached full conversion, the residual was diluted with DCM and washed with saturated sodium bicarbonate aqueous solution (x1), H_2O (x1), and brine (x1), dried over Na_2SO_4 and filtered. The filtrate was concentrated in vacuo and the resulting crude mixture was purified using silica gel column chromatography (Petroleum Ether: EtOAc = 1:1) to afford the desired product **S4** (120 mg, 71 %) as a colourless slurry. Analytical data for **S4** was consistent with those reported previously[3].

The obtained compound **S4** (62 mg, 0.19 mmol) and **S2** (100 mg, 0.227 mmol) together with 4Å molecular sieve (260 mg) were dissolved in anhydrous DCM (2mL). Then the TMSOTf (41 μL, 0.227 mmol) was added. The mixture was stirred at room temperature overnight. The mixture was diluted with DCM (100 mL), then filtered over celite and evaporated under reduced pressure. The residue was purified by silica gel flash chromatography (DCM: MeOH = 15:1) to give the product which is directly used in the next step.

The obtained product was dissolved in mixed solution (MeOH: H_2O = 3:1, 4ml). The pH value was adjusted to 10 with CH_3ONa, keep it stirred at room temperature until full conversion. After neutralize the system with 1M HCl, solvent was removed under reduced pressure and the residue was purified by silica gel flash chromatography (DCM: MeOH = 10:1) to give the product **S5** (32 mg, 30 % over two steps), colourless slurry. ^{1}H NMR (Methanol-d_4, 600 MHz) δ 7.78 (2H, d, J = 7.6 Hz, aromatic), 7.64 (2H, d, J = 7.5 Hz, aromatic), 7.39 (2H, t, J = 7.4 Hz, aromatic), 7.31 (2H, t, J = 7.5 Hz, aromatic), 4.43 (1H, d, J = 8.4 Hz, H-1), 4.35 (2H, d, J = 7.0 Hz), 4.20 (1H, t, J = 7.0 Hz), 3.99-3.90 (2H, m), 3.89-3.86 (1H, m), 3.78 (2H, m), 3.70 (1H, dt, J = 11.2, 4.8 Hz), 3.64-3.59 (3H, m), 3.56-3.47 (3H, m), 3.39 (1H, dt, J = 13.9, 5.4 Hz), 3.12 (2H, t, J = 7.0 Hz), 2.23 (2H, t, J = 7.5 Hz), 2.00 (3H, s, -OAc), 1.64 (2H, p, J = 7.6 Hz), 1.52 (2H, p, J = 7.2 Hz), 1.41-1.33 (2H, m); ^{13}C NMR (151 MHz, CD3OD) δ 174.94, 172.98, 157.49, 143.92, 141.21, 127.46, 126.84, 124.85, 119.63, 101.61 (C-1), 75.07, 71.99, 69.90, 69.35, 68.46, 68.35, 66.33, 61.24, 52.89, 47.14, 40.34, 39.01, 35.72, 29.20, 26.04, 25.28, 22.07. HRMS (ESI): *m/z* calcd for $C_{33}H_{45}N_3O_{10}Na^+$ $[M+Na]^+$: 666.3148; found: 666.3076.

Supplementary References

1. Onyishi, C.U. et al. Toll-like receptor 4 and macrophage scavenger receptor 1 crosstalk regulates phagocytosis of a fungal pathogen. *Nat. Commun.* **14**, 4895 (2023).
2. Sulahian, T. H. et al. Signaling pathways required for macrophage scavenger receptor-mediated phagocytosis: analysis by scanning cytometry. *Respir. Res.* **9**, 59, (2008).
3. Srinivasan, S. et al. Liver-targeted polymeric prodrugs of 8-aminoquinolines for malaria radical cure. *J. Control. Release.* **331**, 213-227, (2021).
