# Supplementary material for: A co-assembly platform engaging macrophage scavenger receptor A for lysosome-targeting protein degradation
Source: Nat Commun. 2024 Feb 23;15:1663. doi: 10.1038/s41467-024-46130-0 (PMC10891067; doi:10.1038/s41467-024-46130-0)
Supplement: Supplementary file 3 — Reporting Summary [file 41467_2024_46130_MOESM3_ESM.pdf]

nature portfolio

Corresponding author(s): Suwei Dong

Last updated by author(s): Jan 27, 2024

Reporting Summary

Nature Portfolio wishes to improve the reproducibility of the work that we publish. This form provides structure for consistency and transparency in reporting. For further information on Nature Portfolio policies, see our Editorial Policies and the Editorial Policy Checklist.

Statistics

For all statistical analyses, confirm that the following items are present in the figure legend, table legend, main text, or Methods section.

n/a	Confirmed	
☐	☒	The exact sample size (n) for each experimental group/condition, given as a discrete number and unit of measurement
☐	☒	A statement on whether measurements were taken from distinct samples or whether the same sample was measured repeatedly
☐	☒	The statistical test(s) used AND whether they are one- or two-sided<br>*Only common tests should be described solely by name; describe more complex techniques in the Methods section.*
☒	☐	A description of all covariates tested
☐	☒	A description of any assumptions or corrections, such as tests of normality and adjustment for multiple comparisons
☐	☒	A full description of the statistical parameters including central tendency (e.g. means) or other basic estimates (e.g. regression coefficient) AND variation (e.g. standard deviation) or associated estimates of uncertainty (e.g. confidence intervals)
☐	☒	For null hypothesis testing, the test statistic (e.g. F, t, r) with confidence intervals, effect sizes, degrees of freedom and P value noted<br>*Give P values as exact values whenever suitable.*
☒	☐	For Bayesian analysis, information on the choice of priors and Markov chain Monte Carlo settings
☒	☐	For hierarchical and complex designs, identification of the appropriate level for tests and full reporting of outcomes
☒	☐	Estimates of effect sizes (e.g. Cohen's d, Pearson's r), indicating how they were calculated

Our web collection on statistics for biologists contains articles on many of the points above.

Software and code

Policy information about availability of computer code

Data collection

Peptide Synthesizer: Instrument-The Liberty Blue Automated Microwave Peptide Synthesizer (CEM), Software-Liberty Blue V1.50.5913.17379; Instrument-CS136X Peptide Synthesizer (CS Bio), Software- CSPEPM V1.1.2;
HPLC purification of compounds: Instrument-Hanbon Sci. & Tech. NP7000 solvent delivery system and Hanbon Sci. & Tech. NU3000 UV detector equipped with Exsil Pure 300 C18 column (10.0 μm, 20 × 250 mm), Software- IChromSolution V3.3.5.0807;
Mass spectroscopy: Instrument-Alliance e2695-SQD mass spectrometer (Waters), Software-Masslynx V4.1;
Flow cytometry: Instrument-Beckman CytoFLEX LX flow cytometer, Software-CytExpert V2.3.1.22;
Confocal microscopy imaging: Instrument-Nikon AXR confocal microscope using a Plan APO ×60, 1.42-NA oil objective with 405-nm violet laser, 488-nm blue laser, 561-nm green laser, and 640-nm red laser, Software- NIS-Elements C
Fluorescence intensity and absorbance measurements: Instrument-Synergy Neo2 Hybrid Multi-Mode Reader (Biotek), Software-Gen5 CHS V3.08;
Dynamic lighting scattering: Instrument-Zetasizer Nano ZSP (Malvern Panalytical), Software-Zetasizer V7.11;
Transmission electron microscopy: Instrument-JEM-1400Plus Electron Microscope (JEOL), Software-Radius V2.0; Instrument- JEM-1400 electron microscope (JEOL), Software-Microscopy Suite V1.7.1;
DNA/RNA characterization: Instrument-NanoDrop™ One/OneC Spectrophotometer (Thermo Fischer);
mRNA expression detection: Instrument-QuantStudio™ 6 Flex, Software-QuantStudio™ real-time PCR instrument operating software V1.7.1;
Protein bands detection: Instrument-Tanon 5200 Imaging System, Software-AllCap ECL;
Tissue slides scanning: Instrument-Zhiyue WS-10, Software-NDP.view 2;
In vivo imaging: Instrument-Perkinelmer IVIS Spectrum, Software-Living Image Software V4.0.

Data analysis

GraphPad Prism V9.4.0, FlowJo V10.8.1, Spectra Manager V2.15.01, NIS-Elements Viewer V5.21, Zetasizer V7.11, ImageJ V1.53c.

For manuscripts utilizing custom algorithms or software that are central to the research but not yet described in published literature, software must be made available to editors and reviewers. We strongly encourage code deposition in a community repository (e.g. GitHub). See the Nature Portfolio guidelines for submitting code & software for further information.

Data

Policy information about availability of data

All manuscripts must include a data availability statement. This statement should provide the following information, where applicable:

- Accession codes, unique identifiers, or web links for publicly available datasets
- A description of any restrictions on data availability
- For clinical datasets or third party data, please ensure that the statement adheres to our policy

All data generated or analyzed in this study are included in this article and its supplementary information files. Source data are provided with this paper.

Research involving human participants, their data, or biological material

Policy information about studies with human participants or human data. See also policy information about sex, gender (identity/presentation), and sexual orientation and race, ethnicity and racism.

Reporting on sex and gender	N/A
Reporting on race, ethnicity, or other socially relevant groupings	N/A
Population characteristics	N/A
Recruitment	N/A
Ethics oversight	N/A

Note that full information on the approval of the study protocol must also be provided in the manuscript.

Field-specific reporting

Please select the one below that is the best fit for your research. If you are not sure, read the appropriate sections before making your selection.

☒ Life sciences ☐ Behavioural & social sciences ☐ Ecological, evolutionary & environmental sciences

For a reference copy of the document with all sections, see nature.com/documents/nr-reporting-summary-flat.pdf

Life sciences study design

All studies must disclose on these points even when the disclosure is negative.

Sample size	For in vitro experiments, typically three experimental replicates were performed based on previous lab experience in order to show standard deviation (SD) and generated acceptable P value in statistic analysis and detect meaningful changes. For in vivo experiments, sample sizes were usually chosen to be five. Sample sizes were based on our previous experience and other publications (J. Clin. Invest. 2020; 130(10): 5180-5196.) The sample size for mouse pharmacokinetic experiments were chosen based on references (Adv. Mater. 2023; 35: 2303831.) with respect to the observed variability differences among groups. They provided enough statistical power to detect the usually strong effects observed in our experiments. All sample sizes are clearly described in the manuscript or the figure legend.
Data exclusions	No data were excluded from the analysis.
Replication	The number of replicates used in the individual experiments are mentioned in the corresponding figures. All the replicates were successful and showed conclusive results.
Randomization	In the animal test, mice were evenly and randomly distributed into experimental groups from the same cohort of mice.
Blinding	No additional blinding was employed for in vitro or in vivo studies in order to make comparisons between specific treatments. Besides, the blinding experimental were not required because experimental conditions could be easily identified from the data itself.

Reporting for specific materials, systems and methods

We require information from authors about some types of materials, experimental systems and methods used in many studies. Here, indicate whether each material, system or method listed is relevant to your study. If you are not sure if a list item applies to your research, read the appropriate section before selecting a response.

Materials & experimental systems

n/a	Involved in the study
☐	☒ Antibodies
☐	☒ Eukaryotic cell lines
☒	☐ Palaeontology and archaeology
☐	☒ Animals and other organisms
☒	☐ Clinical data
☒	☐ Dual use research of concern
☒	☐ Plants

Methods

n/a	Involved in the study
☒	☐ ChIP-seq
☐	☒ Flow cytometry
☒	☐ MRI-based neuroimaging

Antibodies

Antibodies used

PE anti-mouse IL-17A antibody (506903, BioLegend), FC, 1:200
PE anti-mouse CD274 antibody (124307, BioLegend), FC, 1:200
APC anti-mouse CD204 Antibody (154711, BioLegend), FC, 1:200
Rabbit anti-GAPDH antibody (ab181602, Abcam), WB, 1:5000
Mouse anti-GAPDH antibody (60004-1-Ig, Proteintech), WB, 1:5000
Rabbit anti-CD204 antibody (ab151707, Abcam), WB, 1:1000
Mouse anti-PD-L1 antibody (66248-1-Ig, Proteintech), WB, 1:1000
Mouse anti-His-Tag antibody (CW0286, CoWin Biosciences), WB, 1:2000
Rabbit anti-LAMP1 antibody [EPR21026] (ab208943, Abcam), IF, 1:100
Goat Anti-Rabbit IgG H&L (Alexa Fluor® 568) (ab175471, Abcam) , IF 1:200
HRP-conjugated Affinipure Goat Anti-Mouse IgG(H+L) (SA00001-1, Proteintech), WB, 1:5000
HRP-conjugated Affinipure Goat Anti-Rabbit IgG(H+L) (SA00001-2, Proteintech), WB, 1:5000
Phospho-p44/42 MAPK (Erk1/2) (Thr202/Tyr204) (D13.14.4E) XP® Rabbit mAb, (4370T, Cell Signaling Technology), WB, 1:1000
p44/42 MAPK (Erk1/2) (137F5) Rabbit mAb (4695T, Cell Signaling Technology), WB, 1:1000

Validation

All antibodies used were validated by antibody suppliers per quality assurance. Links for each antibody are given below:

PE anti-mouse IL-17A antibody: https://www.biolegend.com/en-us/products/pe-anti-mouse-il-17a-antibody-1633?GroupID=GROUP24. Validated from manufacturer's website and citations therein.

PE anti-mouse CD274 antibody: https://www.biolegend.com/en-us/products/pe-anti-mouse-cd274-b7-h1-pd-l1-antibody-4497. Validated from manufacturer's website and citations therein.

APC anti-mouse CD204 Antibody: https://www.biolegend.com/en-us/products/apc-anti-mouse-cd204-antibody-20744. Validated from manufacturer's website.

Rabbit anti-GAPDH antibody: https://www.abcam.cn/gapdh-antibody-epr16891-loading-control-ab181602.html. Validated from manufacturer's website and citations therein.

Mouse anti-GAPDH antibody:https://www.ptgcn.com/products/GAPDH-Antibody-60004-1-Ig.htm. Validated from manufacturer's website and citations therein.

Rabbit anti-CD204 antibody: https://www.abcam.cn/products/primary-antibodies/cd204-antibody-epr7536-ab151707.html. Validated from manufacturer's website and citations therein.

Mouse anti-PD-L1 antibody: https://www.ptglab.co.jp/products/PD-L1-CD274-Antibody-66248-1-Ig.htm. Validated from manufacturer's website and citations therein.

Mouse anti-His-Tag antibody: https://cwbio.com/goods/index/id/10177. Validated from manufacturer's website.

Rabbit anti-LAMP1 antibody [EPR21026]: https://www.abcam.cn/products/primary-antibodies/lamp1-antibody-epr21026-ab208943.html. Validated from manufacturer's website and citations therein.

Goat Anti-Rabbit IgG H&L (Alexa Fluor® 568): https://www.abcam.cn/products/secondary-antibodies/goat-rabbit-igg-hl-alexa-fluor-568-ab175471.html. Validated from manufacturer's website and citations therein.

HRP-conjugated Affinipure Goat Anti-Mouse IgG(H+L): https://www.ptgcn.com/products/HRP-conjugated-Affinipure-Goat-Anti-Mouse-IgG-H-L-secondary-antibody.htm. Validated from manufacturer's website and citations therein.

HRP-conjugated Affinipure Goat Anti-Rabbit IgG(H+L):https://www.ptgcn.com/Products/HRP-conjugated-Affinipure-Goat-Anti-Rabbit-IgG-H-L-secondary-antibody.htm. Validated from manufacturer's website and citations therein.

Phospho-p44/42 MAPK (Erk1/2) (Thr202/Tyr204) (D13.14.4E) XP® Rabbit mAb: https://www.cellsignal.cn/products/primary-antibodies/phospho-p44-42-mapk-erk1-2-thr202-tyr204-d13-14-4e-xp-174-rabbit-mab/4370

p44/42 MAPK (Erk1/2) (137F5) Rabbit mAb: https://www.cellsignal.cn/products/primary-antibodies/p44-42-mapk-erk1-2-137f5-rabbit-mab/4695

Eukaryotic cell lines

Policy information about cell lines and Sex and Gender in Research

Cell line source(s)	RAW264.7(1101MOU-PUMC000146) and HepG2(1101HUM-PUMC000035) cells were obtained from the Cell Resource Center, Peking Union Medical College (PCRC). U-118MG cells were purchased from iCell (Cat# iCell-h217). HaCaT cells were purchased from MeisenCTCC (Cat#CTCC-OO2-OO12). DC2.4 and A549 cells were generous gift from Prof. Peng Chen's Lab and Prof. Houhua Li's Lab respectively at Peking University.
Authentication	Cell lines have not been subjected to additional authentication.
Mycoplasma contamination	All cell lines regularly tested negative for mycoplasma contamination.
Commonly misidentified lines (See ICLAC register)	No commonly misidentified cell lines were used in this study.

Animals and other research organisms

Policy information about studies involving animals; ARRIVE guidelines recommended for reporting animal research, and Sex and Gender in Research

Laboratory animals	8 to10-week-old female BALB/c mice were used for the in vivo experiments. All mice were housed in SPF animal facility (light/dark cycle: 12h/12h , temperature: 20-26°C, humidity: 40-70%) with adlibitum access to food and water.
Wild animals	No wild animals were used.
Reporting on sex	Only female mice participated in this experimental study.
Field-collected samples	The study did not involve field-collected samples.
Ethics oversight	All animal experiments in this work were performed under the Guide for Care and Use of Laboratory Animals and approved by the Experimental Animal Welfare Ethics Subcommittee of Peking University Biomedical Ethics Committee (LA2022226).

Note that full information on the approval of the study protocol must also be provided in the manuscript.

Flow Cytometry

Plots

Confirm that:

☒ The axis labels state the marker and fluorochrome used (e.g. CD4-FITC).

☒ The axis scales are clearly visible. Include numbers along axes only for bottom left plot of group (a 'group' is an analysis of identical markers).

☒ All plots are contour plots with outliers or pseudocolor plots.

☒ A numerical value for number of cells or percentage (with statistics) is provided.

Methodology

Sample preparation	For IL-17A uptake experiment, cells were incubated with indicated treatments for indicated time. After washing with PBS for three times, the cells were transferred to the tubes, fixed and permeabilized on ice by 4% polyformaldehyde and the permeabilization buffer respectively. Then, the cells were treated with the PE anti-mouse IL-17A antibody for 30 minutes on ice. Finally, the cells were washed for three times for the flow cytometry analysis. For surface staining experiments, cells were incubated with indicated treatments for indicated time. After washing with PBS for three times, the cells were transferred to the tubes and treated with the PE anti-mouse CD274 antibody or APC anti-CD204 antibody for 30 minutes on ice. Finally, the cells were washed for three times for the flow cytometry analysis.
Instrument	Flow experiments were performed using an Beckman CytoFLEX LX flow cytometer.
Software	CytExpert V2.3.1.22; FlowJo V10.8.1; GraphPad Prism V9.4.0.
Cell population abundance	N/A - cell sorting was not performed.
Gating strategy	Debris was gated out by the FSC and SSC area, and single cells were gated on FSC-H and FSC-Width.

☒ Tick this box to confirm that a figure exemplifying the gating strategy is provided in the Supplementary Information.
